# Prevalence and incidence of comorbidities in patients with atopic dermatitis, psoriasis, alopecia areata, and vitiligo using a Japanese claims database

**DOI:** 10.1111/1346-8138.17643

**Published:** 2025-02-07

**Authors:** Yue Ma, Motohiko Chachin, Tomohiro Hirose, Kouki Nakamura, Nanzhi Shi, Shintaro Hiro, Shinichi Imafuku

**Affiliations:** ^1^ Pfizer Japan Inc. Tokyo Japan; ^2^ Pfizer R&D Japan Tokyo Japan; ^3^ Department of Dermatology Fukuoka University Fukuoka Japan

**Keywords:** alopecia areata, atopic dermatitis, Japan, psoriasis, vitiligo

## Abstract

Atopic dermatitis, psoriasis, alopecia areata, and vitiligo have been associated with comorbid conditions, including infections, malignancies, and cardiovascular diseases. This study evaluated the prevalence and incidence rates of these comorbidities in patients from Japan. This retrospective cohort study used data collected from the JMDC claims database between June 2013 and December 2020. Patients with a diagnosis of atopic dermatitis, psoriasis, alopecia areata, or vitiligo were matched (1:1) by age, sex, and index month with individuals with no claims records for atopic dermatitis, psoriasis, alopecia areata, or vitiligo diagnosis. Data included 691 338, 51 988, 43 692, and 8912 patients in the atopic dermatitis, psoriasis, alopecia areata, and vitiligo cohorts, respectively, and matched controls. The most prevalent comorbidities in the atopic dermatitis cohort versus matched controls included allergic rhinitis (47% vs 37%), conjunctivitis (33% vs 23%), asthma (27% vs 20%), viral infection (22% vs 15%), and acne (11% vs 3%). Incidence rates per 100 000 person‐years of comorbidities in the atopic dermatitis cohort versus matched controls were: venous thromboembolism, 51.4 (95% confidence interval [CI], 48.3–54.7) versus 31.7 (95% CI, 29.2–34.2); lymphoma, 13.8 (95% CI,12.2–15.6) versus 5.7 (95% CI, 4.7–6.8); cutaneous T‐cell lymphoma, 1.6 (95% CI, 1.1–2.2) versus 0.1 (95% CI, 0.0–0.4); and herpes zoster, 740.9 (95% CI, 728.8–753.1) versus 397.6 (95% CI, 388.9–406.6). Similar trends were observed in the psoriasis versus nonpsoriasis cohorts, with 95% CIs mostly overlapping for alopecia areata and vitiligo cohorts versus controls. Overall, patients from Japan with dermatologic diseases have a higher prevalence and incidence of certain health conditions, particularly venous thromboembolism, lymphoma, and infections in patients with atopic dermatitis and psoriasis, compared with individuals without these dermatologic diseases.

## 1 INTRODUCTION

Chronic inflammatory or autoimmune dermatologic diseases, including atopic dermatitis (AD), psoriasis, alopecia areata (AA), and vitiligo, have been associated with certain comorbidities, including other dermatologic conditions, cardiovascular diseases (CVDs), malignancies, and infections.^1^, ^2^, ^3^, ^4^, ^5^, ^6^, ^7^, ^8^ Previous population‐based studies in the United Kingdom, United States, and some Asian and European countries found an increased risk of CVDs, including stroke, myocardial infarction, heart failure, or cardiovascular death; cancers, including lymphoma, melanoma, or nonmelanoma skin cancer (NMSC); and infections in patients with AD,^1^, ^9^, ^10^, ^11^, ^12^ psoriasis,^5^, ^13^, ^14^, ^15^, ^16^ AA,^3^ and vitiligo.^17^ In patients with AD or psoriasis, the risk of CVD, lymphoma, and infections, including herpes zoster, appears to increase with disease severity.^1^, ^7^, ^9^, ^10^, ^14^, ^15^ Comorbidities such as depression, anxiety, and other psychiatric or psychosocial conditions are also common in patients with dermatologic diseases, indicating the profound negative impact of dermatologic conditions on quality of life.^2^, ^18^, ^19^, ^20^, ^21^, ^22^

The incidence and risk of comorbidities in patients with dermatologic diseases may differ depending on geographical location, race, or ethnicity. There is conflicting evidence from studies of patients with vitiligo in European and Asian countries reporting that the risk of malignancies such as melanoma and NMSC may be increased,^17^ decreased,^23^ or remain unchanged,^24^, ^25^ suggesting differences depending on geographical location. Patients from Taiwan with AA had a higher risk of melanoma, lymphoma, breast cancer, kidney cancer, and urinary bladder cancer, and a lower risk of overall cancers and NMSC compared with individuals without AA; whereas, in patients with AA from Korea the risk of overall cancers was slightly higher and the risk of lymphoma was comparable to that in individuals without AA.^26^, ^27^ Comparative analysis of age‐standardized incidence rates (IRs) from the Cancer Incidence in Five Continents database suggested that the rate of melanoma was higher in Japanese individuals in the United States compared with Japanese individuals in Japan.^28^

The onset of AD, psoriasis, AA, and vitiligo are triggered by immunological mechanisms; AD and psoriasis are predominantly Th2‐ and Th17‐mediated, respectively, while AA and vitiligo are predominantly Th1‐mediated.^29^, ^30^, ^31^ Due to the immune response mechanisms associated with these conditions, interest in the use of immune response–targeting treatments has increased.^29^, ^32^, ^33^ Current treatment strategies for dermatologic diseases include the use of corticosteroids, immunosuppressive agents, phototherapy, and biological agents.^29^, ^34^, ^35^, ^36^, ^37^ Consequently, individuals with dermatological diseases may be at an increased risk of comorbidities associated with the use of corticosteroids and immunosuppressive drugs as a result of their immunosuppressive effects.^34^, ^37^, ^38^ Treatments for psoriasis targeting cytokines involved in immune response (tumor necrosis factor α, interleukin [IL]‐ 17A, and IL‐23) have been shown to decrease the risk of psoriasis‐associated comorbidities, such as myocardial infarction; however, there is a risk of associated infections.^29^, ^38^

Few real‐world studies have investigated the prevalence and IRs of comorbidities in patients with dermatologic diseases in Japan. A recent retrospective cross‐sectional study using data from the JMDC database, one of the largest claims databases in Japan, showed that atopic diseases, including AD, asthma, and allergic rhinitis, were the most prevalent comorbidities in patients with AA.^39^ To better inform clinical management and support patient care, this retrospective real‐world study aimed to estimate the prevalence and IRs of comorbidities, including CVDs, malignancies, infections, and dermatologic and allergic conditions, in patients from Japan with AD, psoriasis, AA, and vitiligo using the JMDC claims database.

## 2 METHODS

### 2.1 Data source and study design

This retrospective cohort study used data collected from the JMDC claims database. The JMDC manages claims data from Japan‐based health insurance associations for employees of mid‐ to large‐size companies and their families. This longitudinal database includes inpatient and outpatient data and pharmacy claims, and each patient can be tracked by a consistent unique identifier. Based on a feasibility assessment in May 2019, data in the JMDC represented 6.1% of the Japanese population. This study used an anonymized structured database that did not contain any personal information that allows for identification of individuals. According to the Ethical Guidelines for Human Life Science and Medical Research in Japan, informed consent is not required for studies using anonymized data. Institutional review board approval and ethics committee approval were not required. This study was conducted in accordance with legal and regulatory requirements, as well as with scientific purpose, value, and rigor, and followed generally accepted research practices as described in the Guidelines for Good Pharmacoepidemiology Practices issued by the International Society for Pharmacoepidemiology and Good Practices for Outcomes Research issued by the International Society for Pharmacoeconomics and Outcomes Research.

### 2.2 Participants and setting

Data were collected from the JMDC claims database between June 2013 and December 2020. Participants were eligible to be included in one of four dermatologic disease cohorts (AD, psoriasis, AA, or vitiligo) if they had at least two records of definitive diagnoses in different months between June 2014 and December 2020 of AD (*International Classification of Diseases, Tenth Revision* [*ICD‐10*], code L20), psoriasis (*ICD‐10* code L40), AA (*ICD‐10* code L63), or vitiligo (*ICD‐10* code L80), with at least one record at least 12 months after database entry. In the AD cohort, patients were required to have at least two records of treatment, including topical corticosteroids, topical calcineurin inhibitors, dupilumab, or other AD therapies, administered within 1 month of the date of definitive AD diagnosis. Patients with psoriasis were required to have at least two records of treatment, including vitamin D3 topical drugs, topical corticosteroids, phosphodiesterase 4 inhibitors, or other psoriasis therapies administered within 1 month of the date of definitive psoriasis diagnosis.

Participants were eligible for inclusion in non‐AD, nonpsoriasis, non‐AA, or nonvitiligo control cohorts if they had no claims with *ICD‐10* codes for AD, psoriasis, AA, or vitiligo, respectively, during the study period. Participants in the AD, psoriasis, AA, and vitiligo cohorts were matched 1:1 with the non‐AD, nonpsoriasis, non‐AA, and nonvitiligo cohorts, respectively. Matching was performed to summarize disease characteristics, with the index month, age at index month (up to +1 year; i.e., the same age or 1 year older), and sex used as matching keys. In this study, sex was defined as a dichotomous variable (male or female). JMDC patient records reflect sex assigned at birth unless changed upon request by the insured individual.

For patients whose first record of diagnosis was at least 1 year after database entry (patients with a new diagnosis), the index month was defined as the first record of diagnosis or treatment of AD, psoriasis, AA, or vitiligo. The baseline period for this subgroup was defined as the 1 year before the first record of diagnosis (Figure S1a). For patients whose first record of diagnosis was less than 1 year after initial database entry (patients with prevalent diagnosis), the index month was defined as 12 months after database entry, and the baseline period was defined as the 1 year from database entry through the index month (Figure S1b). Individuals with less than 1 year of data before the index month were excluded from the study. The follow‐up period started on the first day after the end of the index month and terminated at the earlier date of either the loss of enrollment in insurance policy or 6 months after the final diagnosis or treatment. Data collected included age, sex, diagnoses, clinical procedures, prescription drugs, cancer management, and treatment costs.

### 2.3 Outcomes

Prevalent health conditions in patients with AD, psoriasis, AA, and/or vitiligo were evaluated in comparison with matched controls and identified from database records during the baseline period using *ICD‐10* codes, as shown in Table S1, in the following categories: allergic diseases, infections, mental health disorders, respiratory diseases, malignancies, CVDs, metabolic disorders, dermatosis, autoimmune diseases, and other diseases. Participants had to have at least one record of a definitive diagnosis using *ICD‐10* codes for a condition, except for AD, psoriasis, AA, and vitiligo (at least two definitive diagnoses required). Prevalence (percentage) was defined as follows: (number of patients with each comorbidity/total number of patients in each cohort) multiplied by 100.

Patients with dermatologic diseases, including AD, psoriasis, and AA, treated with systemic therapies may have a higher risk of developing certain health conditions, including cardiovascular events or infections.^40^, ^41^, ^42^ IRs were determined for outcomes of special interest diagnosed during the follow‐up period for each of the AD, psoriasis, AA, and vitiligo cohorts, as well as their respective matched control cohorts, including major cardiovascular events, venous thromboembolism (VTE) events, malignancies, herpes zoster, and tuberculosis. Major cardiovascular events were defined as ischemic heart diseases and stroke with hospitalization, with at least one record of definitive diagnosis using *ICD‐10* codes listed in Table S1. VTE was identified based on a record of diagnosis using *ICD‐10* codes for VTE (Table S1) and a record of prescription, within the same month but not 2 months prior, for heparin, fondaparinux, urokinase, tissue plasminogen activator (monteplase), oral anticoagulant, warfarin, edoxaban, rivaroxaban, or apixaban; inferior vena cava filter placement; or thrombus removal. Malignancies, including or excluding NMSC, were defined based on one record of diagnosis within a claim month and a procedure record of cancer management within the same claim month or at least one other claim month, using *ICD‐10* codes listed in Table S1.

Herpes zoster and tuberculosis events were defined by at least one record of definitive diagnosis using *ICD‐10* codes (Table S1), and a record of treatment with antiviral therapy within less than 2 months after diagnosis, with or without a record of herpes simplex. Tuberculosis events were defined by at least one record of definitive diagnosis using *ICD‐10* codes (Table S1), and at least two records of treatment 1 month after diagnosis. IRs were determined in the overall population in each cohort for the combined patients with prevalent and new diagnosis. Patients were excluded from the IR analysis of major cardiovascular events, VTE events, or malignancies if these outcomes occurred during the baseline period. IRs (number of persons with events per 100 000 person‐years [PY]) were calculated as follows: (number of new persons who experienced an event/the sum of person‐month) multiplied by 100 000 multiplied by 12.

IRs were also assessed in subcohorts classified by age (0–9, 10–19, 20–29, 30–39, 40–49, 50–59, ≥60 years). IRs associated with a specific drug product were not assessed.

### 2.4 Statistical analysis

This study was not designed for hypothesis testing, and, therefore, sample size calculations were not applicable. Patient baseline characteristics were summarized using descriptive statistics. For categorical variables, proportions of patients were analyzed, and for comorbidity variables, prevalence (percentage) was analyzed. For count variables, the number of patients who experienced the event per the sum of the observed person‐time at risk, i.e., IRs per 100 000 PY and their 95% CIs are presented. Two‐sided 95% CIs for IRs were calculated based on the Poisson distribution and its relation to the chi‐squared distribution.

## 3 RESULTS

### 3.1 Baseline demographics and disease characteristics

**FIGURE 1:**
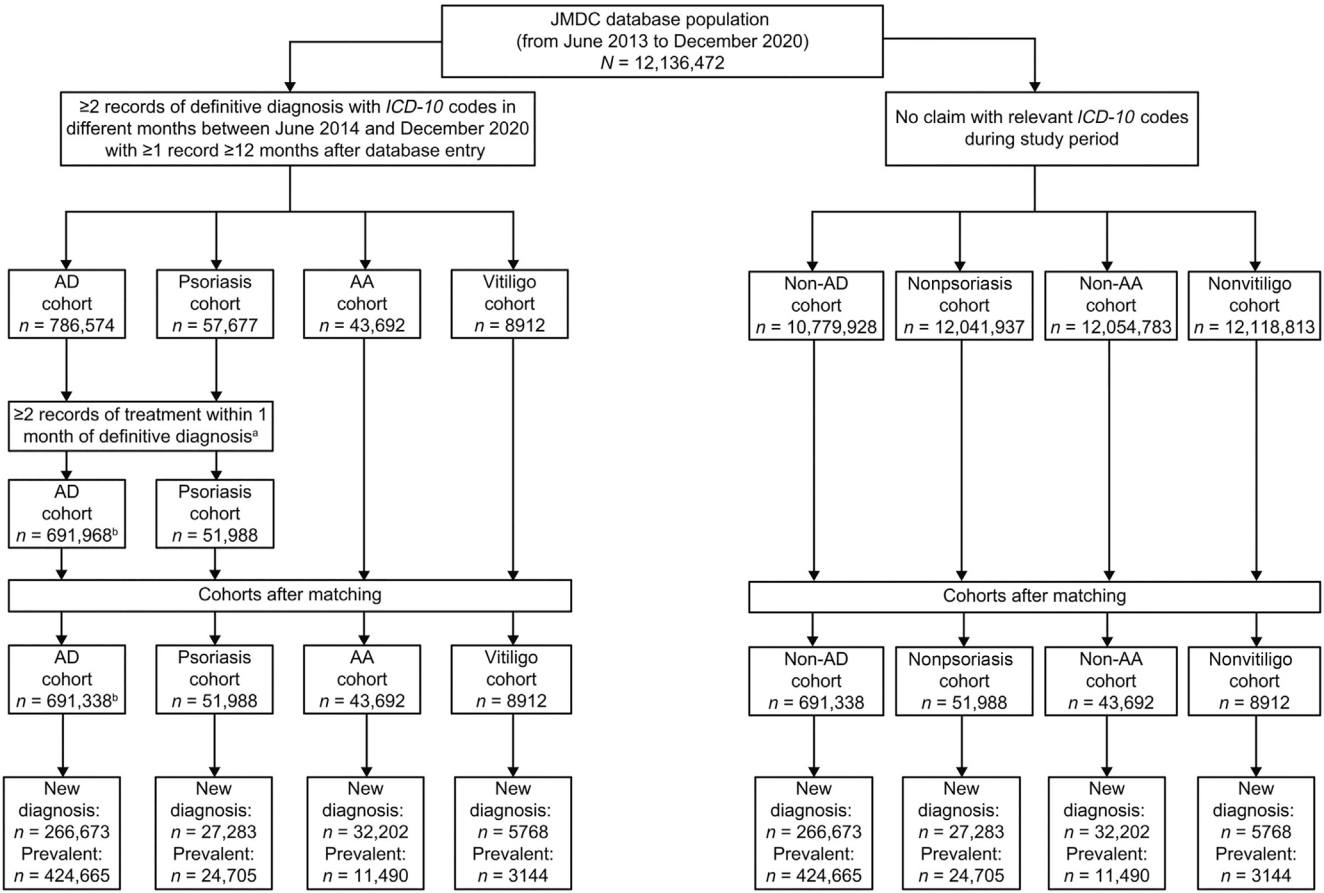
Cohort screening. The AD, psoriasis, AA, and vitiligo cohorts were matched in a 1:1 ratio with non‐AD, nonpsoriasis, non‐AA, and nonvitiligo control cohorts by sex, age, and index month. ^a^In the AD cohort, patients were required to have at least two records of treatment, including topical corticosteroids, topical calcineurin inhibitors, dupilumab, or other AD therapies, administered within 1 month of the date of definitive AD diagnosis. Patients with psoriasis were required to have at least two records of treatment, including vitamin D3 topical drugs, topical corticosteroids, phosphodiesterase 4 inhibitors, or other psoriasis therapies administered within 1 month of the date of definitive psoriasis diagnosis. ^b^Patients in the AD cohort who did not have a non‐AD–matching individual were excluded from the final AD cohort population analyzed. AA, alopecia areata; AD, atopic dermatitis; *ICD‐10*, *International Classification of Diseases, Tenth Revision*.

Overall, data were collected for analysis from 12 136 472 individuals from the JDMC database between June 2013 and December 2020 (Figure 1). Of these, 691 338, 51 988, 43 692, and 8912 patients were included in the AD, psoriasis, AA, and vitiligo cohorts, respectively, and an equal number of individuals were enrolled in the respective matched control cohorts. In the AD cohort, 61.4% and 38.6% of patients were included in the prevalent and new diagnosis patient subgroups, respectively, 47.5% and 52.5% in the psoriasis cohort, 26.3% and 73.7% in the AA cohort, and 35.3% and 64.7% in the vitiligo cohort (Table 1).

**TABLE 1:** Patient demographics and healthcare utilization patterns during the baseline period.

Characteristics	AD		Psoriasis		AA		Vitiligo	
	AD cohort (*n* = 691 338)	Non‐AD cohort (*n* = 691 338)	Psoriasis cohort (*n* = 51 988)	Nonpsoriasis cohort (*n* = 51 988)	AA cohort (*n*= 43 692)	Non‐AA cohort (*n* = 43 692)	Vitiligo cohort (*n* = 8912)	Nonvitiligo cohort (*n* = 8912)
Prevalent diagnosis, *n* (%)^a^	424 665 (61.4)	424 665 (61.4)	24 705 (47.5)	24 705 (47.5)	11 490 (26.3)	11 490 (26.3)	3144 (35.3)	3144 (35.3)
New diagnosis, *n* (%)^a^	266 673 (38.6)	266 673 (38.6)	27 283 (52.5)	27 283 (52.5)	32 202 (73.7)	32 202 (73.7)	5768 (64.7)	5768 (64.7)
Age categories, *n* (%), years								
0–9	273 123 (39.5)	273 123 (39.5)	1899 (3.7)	1899 (3.7)	3219 (7.4)	3219 (7.4)	1917 (21.5)	1917 (21.5)
10–19	109 054 (15.8)	109 054 (15.8)	2224 (4.3)	2224 (4.3)	4720 (10.8)	4720 (10.8)	1252 (14.0)	1252 (14.0)
20–29	95 179 (13.8)	95 179 (13.8)	3678 (7.1)	3678 (7.1)	4905 (11.2)	4905 (11.2)	687 (7.7)	687 (7.7)
30–39	87 738 (12.7)	87 738 (12.7)	7667 (14.7)	7667 (14.7)	8810 (20.2)	8810 (20.2)	1174 (13.2)	1174 (13.2)
40–49	73 292 (10.6)	73 292 (10.6)	14 021 (27.0)	14 021 (27.0)	11 320 (25.9)	11 320 (25.9)	1566 (17.6)	1566 (17.6)
50–59	39 081 (5.7)	39 081 (5.7)	14 327 (27.6)	14 327 (27.6)	7946 (18.2)	7946 (18.2)	1426 (16.0)	1426 (16.0)
≥60	13 871 (2.0)	13 871 (2.0)	8712 (16.8)	8712 (16.8)	2772 (6.3)	2772 (6.3)	890 (10.0)	890 (10.0)
Age at index month, mean (SD), years	20.4 (17.7)	20.9 (17.7)	44.9 (15.2)	45.4 (15.2)	37.2 (16.2)	37.7 (16.2)	32.5 (20.8)	33.0 (20.8)
Sex, *n* (%)								
Male	352 305 (51.0)	352 305 (51.0)	29 690 (57.1)	29 690 (57.1)	19 059 (43.6)	19 059 (43.6)	4135 (46.4)	4135 (46.4)
Female	339 033 (49.0)	339 033 (49.0)	22 298 (42.9)	22 298 (42.9)	24 633 (56.4)	24 633 (56.4)	4777 (53.6)	4777 (53.6)
Follow‐up period, mean (SD), years	2.9 (1.9)	2.9 (1.9)	2.5 (1.8)	2.5 (1.8)	1.5 (1.3)	1.5 (1.3)	1.8 (1.4)	1.8 (1.4)
Medical resources, mean (SD)^b^								
Insurance points for treatment^c^	19981.3 (69743.2)	11179.6 (44852.5)	33333.3 (95949.1)	15334.5 (70446.3)	19899.2 (69984.1)	11584.2 (36392.3)	21365.9 (58360.7)	13944.2 (50377.0)
Insurance points for treatment^c^ (per month)	1665.1 (5811.9)	931.7 (3737.7)	2777.8 (7995.8)	1277.9 (5870.5)	1658.3 (5832.0)	965.4 (3032.7)	1780.5 (4863.4)	1162.1 (4198.1)
Visits to medical facilities (dermatology)	5.2 (10.2)	1.9 (6.9)	7.0 (13.3)	2.2 (7.7)	5.3 (13.1)	2.1 (7.2)	7.7 (13.6)	2.4 (7.9)
Visits to medical facilities (per month; dermatology)	0.4 (0.9)	0.2 (0.6)	0.6 (1.1)	0.2 (0.6)	0.5 (1.1)	0.2 (0.6)	0.6 (1.1)	0.2 (0.7)
Visits to medical facilities (all departments)	16.1 (17.1)	10.0 (13.6)	17.0 (21.5)	9.7 (15.6)	14.3 (20.3)	8.7 (13.3)	18.3 (20.7)	10.5 (15.8)
Visits to medical facilities (per month; all departments)	1.4 (1.4)	0.8 (1.1)	1.4 (1.8)	0.8 (1.3)	1.2 (1.7)	0.7 (1.1)	1.5 (1.7)	0.9 (1.3)
Treatments, *n* (%)^b^								
Corticosteroids	92 713 (13.4)	45 717 (6.6)	7639 (14.7)	3361 (6.5)	5236 (12.0)	3009 (6.9)	958 (10.7)	654 (7.3)
Dose (SD) mg/day^d^	16.3 (24.6)	17.3 (23.5)	14.4 (19.5)	17.6 (25.4)	15.9 (21.0)	18.1 (24.4)	17.4 (26.1)	18.2 (26.0)
Phototherapy	19 482 (2.8)	4485 (0.6)	4228 (8.1)	423 (0.8)	2506 (5.7)	401 (0.9)	1677 (18.8)	78 (0.9)
Biologics	7460 (1.1)	4703 (0.7)	1751 (3.4)	611 (1.2)	515 (1.2)	385 (0.9)	121 (1.4)	80 (0.9)
Immunosuppressive drug	4337 (0.6)	1092 (0.2)	3197 (6.1)	187 (0.4)	380 (0.9)	131 (0.3)	69 (0.8)	26 (0.3)

Abbreviations: AA, alopecia areata; AD, atopic dermatitis; SD, standard deviation.

^a^ For patients whose first record of diagnosis was at least 1 year after database entry (new diagnosis subgroup), the index month was defined as the first record of diagnosis or treatment of AD, psoriasis, AA, or vitiligo. For patients whose first record of diagnosis was 1 year or less after initial database entry (prevalent diagnosis subgroup), the index month was defined as 12 months after database entry, and the baseline period was defined as the 1 year from database entry through the index month.

^b^ Medical resources and treatment were determined during the baseline period and the follow‐up period. The baseline period was defined as a 1‐year interval prior to and including the index month. For each initiation of an exposure of interest, the follow‐up started on the first day of the next month after the index month. The follow‐up period ended at the earlier date of either the loss of enrollment in insurance policy or 6 months after the final diagnosis or treatment. Use of oral corticosteroids, phototherapy, biologics, or immunosuppressive drugs (AYC code L04) were considered when patients had at least one prescription for these drugs (or treatment for phototherapy) during the baseline period and follow‐up period.

^c^ One insurance point = 10 Japanese yen.

^d^ The oral corticosteroid dose was converted to the equivalent prednisolone dose (mg/day).

Greater proportions of patients were male in the psoriasis cohort and female in the AA and vitiligo cohorts; proportions of males and females were comparable in the AD cohort (Table 1). The mean age at the index month ranged from 20.4 to 45.4 years among all cohorts (Table 1). The mean age at index month was 20.4 years (standard deviation [SD], 17.7 years), 44.9 years (SD, 15.2 years), 37.2 years (SD, 16.2 years), and 32.5 years (SD, 20.8 years) in the AD, psoriasis, AA, and vitiligo cohorts, respectively. The mean duration of the follow‐up period was 2.9 years (SD, 1.9 years), 2.5 years (SD, 1.8 years), 1.5 years (SD, 1.3 years), and 1.8 years (SD, 1.4 years) in the AD, psoriasis, AA, and vitiligo cohorts, respectively.

Patients diagnosed with AD, psoriasis, AA, and vitiligo consistently had greater insurance utilization and higher frequency of visits to medical facilities compared with matched controls (Table 1). Corticosteroids were the most common treatment in the AD (13.4%), psoriasis (14.7%), and AA (12.0%) cohorts. Patients with vitiligo were most frequently treated with phototherapy (18.8%).

### 3.2 Prevalence of comorbidities

**FIGURE 2:**
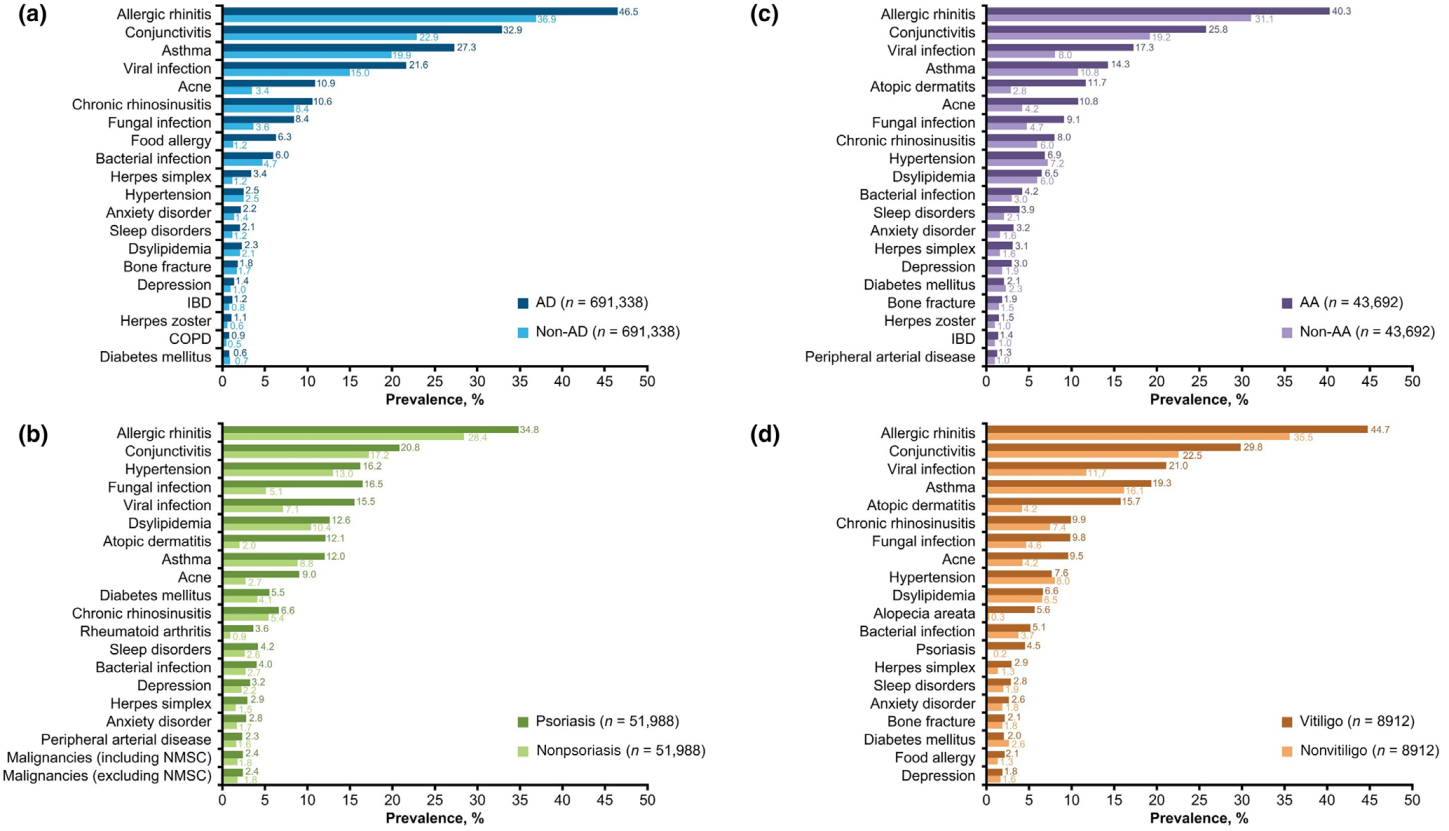
Prevalence of the 20 most frequent comorbidities during the baseline period in matched (a) AD and non‐AD, (b) psoriasis and nonpsoriasis, (c) AA and non‐AA, and (d) vitiligo and nonvitiligo cohorts. AA, alopecia areata; AD, atopic dermatitis; COPD, chronic obstructive pulmonary disease; IBD, inflammatory bowel disease (including ulcerative colitis and Crohn disease); NMSC, nonmelanoma skin cancer.

A total of 54 different comorbidities were identified among all cohorts during the baseline period. The 20 most frequently occurring comorbidities in each disease cohort are shown in Figure 2. The prevalence of allergic diseases was generally higher in the disease cohorts compared with the matched controls for allergic rhinitis (AD: 47% vs 37%; psoriasis: 35% vs 28%; AA: 40% vs 31%; and vitiligo: 45% vs 36%), conjunctivitis (AD: 33% vs 23%; psoriasis: 21% vs 17%; AA: 26% vs 19%; and vitiligo: 30% vs 22%), and asthma (AD: 27% vs 20%; psoriasis: 12% vs 9%; AA: 14% vs 11%; and vitiligo: 19% vs 16%) (Figure 2a–d).

Similar trends were observed for dermatologic diseases and infections. Notably, acne was more prevalent in the disease cohorts compared with the matched controls (AD: 11% vs 3%; psoriasis: 9% vs 3%; AA: 11% vs 4%; and vitiligo: 10% vs 4%) (Figure 2a–d). Comorbid dermatologic diseases were also more prevalent in the disease cohorts compared with the matched controls, including AD in the psoriasis cohort (12% vs 2%), AA cohort (12% vs 3%), and vitiligo cohort (16% vs 4%), and psoriasis (5% vs 0%) and AA (6% vs 0%) in the vitiligo cohort (Figure 2b–d). Prevalence of psoriasis was low in the AD and AA cohorts (1% vs 1%), with a similar trend evident for AA in the psoriasis and AD cohorts (1% vs 1%). Viral infections (AD: 22% vs 15%; psoriasis: 16% vs 7%; AA: 17% vs 8%; and vitiligo: 21% vs 12%) and fungal infections (AD: 8% vs 4%; psoriasis: 17% vs 5%; AA: 9% vs 5%; and vitiligo: 10% vs 5%) were also more prevalent in the disease cohorts compared with the matched controls.

For malignancies, mental health disorders, CVDs, metabolic disorders, dermatosis, autoimmune diseases, and other diseases such as bone fracture and dyslipidemia, prevalence was generally similar between AD, psoriasis, AA, and vitiligo cohorts and their matched controls (Figure 2a–d).

### 3.3 Incidence of major cardiovascular and VTE events

**FIGURE 3:**
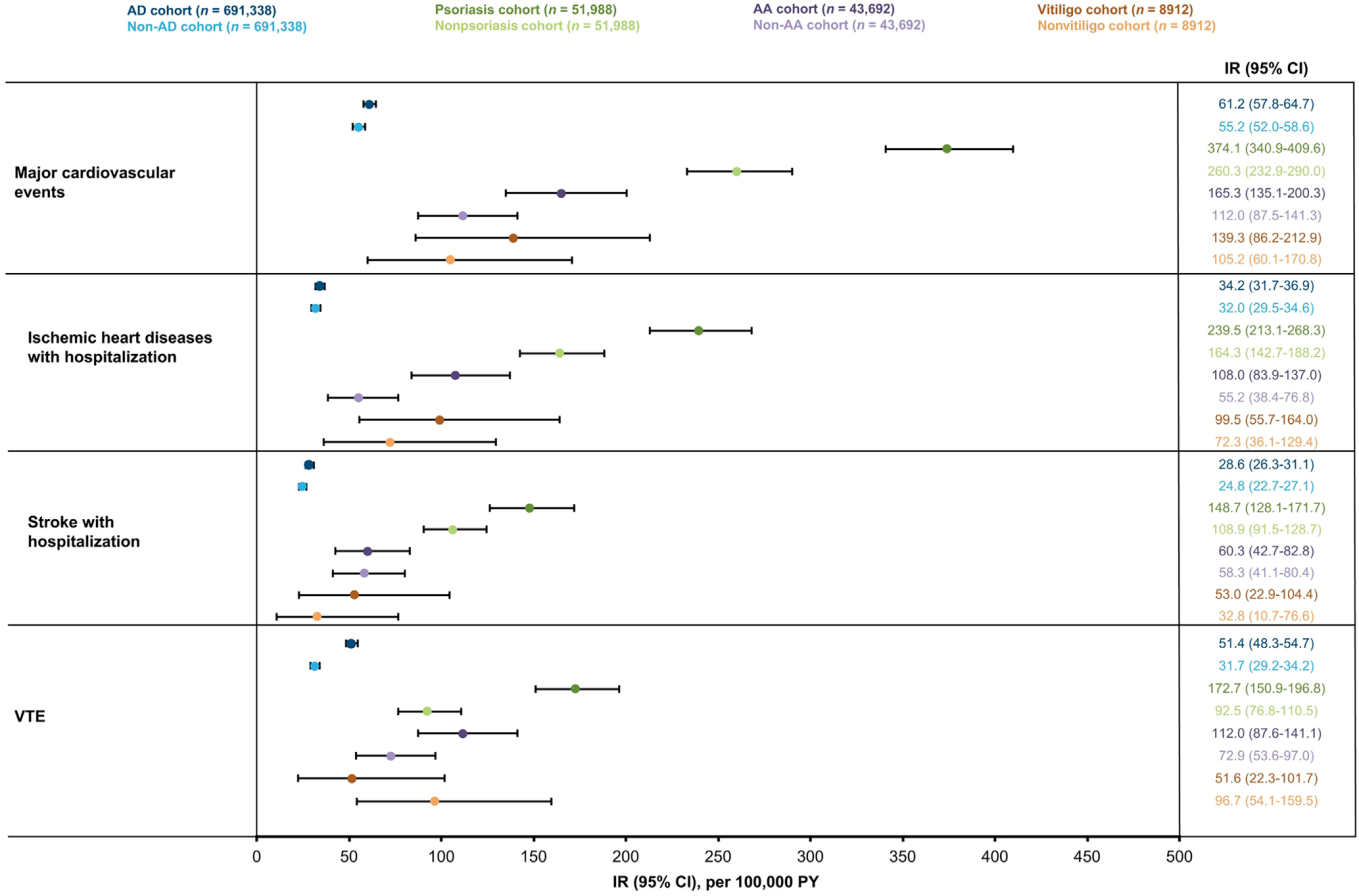
IRs of major cardiovascular events and VTE events in patients with AD, psoriasis, AA, and vitiligo compared with index month‐, age‐, and sex‐matched controls during the follow‐up period. Ischemic heart diseases were diagnosed using *ICD‐10* code I20‐25 (i.e., any of ischemic heart disease diagnosis codes [acute coronary syndrome and coronary artery disease]), and stroke was diagnosed using *ICD‐10* code I60‐64 (i.e., any stroke diagnosis code). AA, alopecia areata; AD, atopic dermatitis; CI, confidence interval; *ICD‐10*, *International Classification of Diseases, Tenth Revision*; IR, incidence rate; PY, person‐years; VTE, venous thromboembolism.

The IRs of major cardiovascular events, including ischemic heart disease and stroke leading to hospitalization during the follow‐up period, were comparable between the AD cohort and the non‐AD control cohort; however, the IR of VTE was higher in the AD cohort compared with the control cohort (IR/100 000 PY, 51.4 [95% CI, 48.3–54.7] vs 31.7 [95% CI, 29.2–34.2], respectively; Figure 3). IRs of major cardiovascular events (IR/100 000 PY, 374.1 [95% CI, 340.9–409.6] vs 260.3 [95% CI, 232.9–290.0]), ischemic heart disease leading to hospitalization (239.5 [95% CI, 213.1–268.3] vs 164.3 [95% CI, 142.7–188.2]), stroke leading to hospitalization (148.7 [95% CI, 128.1–171.7] vs 108.9 [95% CI, 91.5–128.7]), and VTE (172.7 [95% CI, 150.9–196.8] vs 92.5 [95% CI, 76.8–110.5]) were higher in the psoriasis cohort compared with the nonpsoriasis control cohort.

The IR of ischemic heart disease leading to hospitalization was higher in the AA cohort compared with the non‐AA control cohort (IR/100 000 PY, 108.0 [95% CI, 83.9–137.0] vs 55.2 [95% CI, 38.4–76.8], respectively). The IRs of major cardiovascular events overall and of stroke leading to hospitalization, as well as of VTE events, were numerically higher in the AA cohort compared with the non‐AA control cohort; however, 95% CIs were widely overlapping. Similarly, the IRs of overall major cardiovascular events, ischemic heart disease, and stroke leading to hospitalization were numerically higher in the vitiligo cohort compared with the nonvitiligo control cohort, with overlapping 95% CIs (Figure 3).

### 3.4 Incidence of malignancies

**FIGURE 4:**
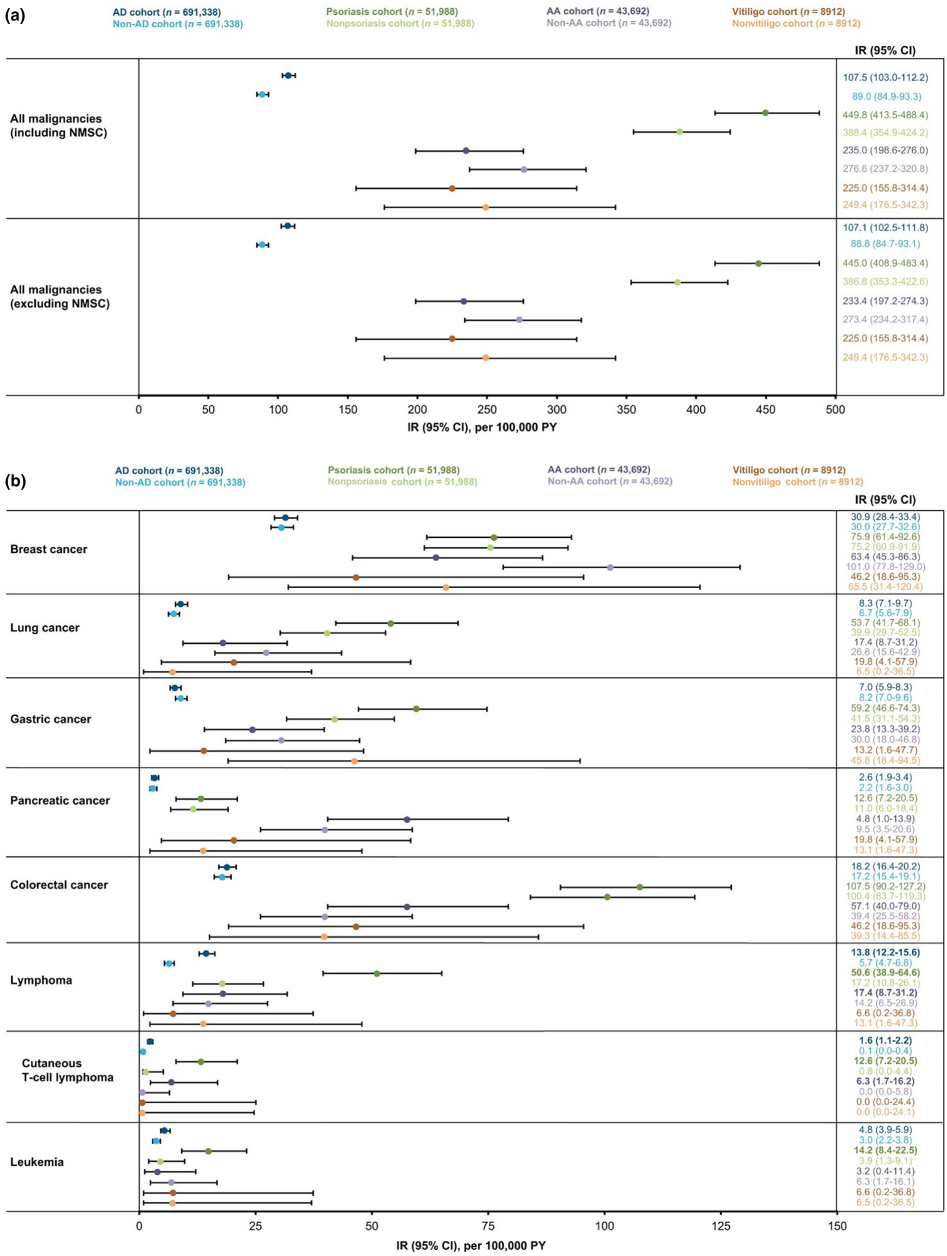
IRs of (a) all malignancies, including or excluding NMSC, and (b) malignancy subtypes, in patients with AD, psoriasis, AA, and vitiligo compared with index month‐, age‐, and sex‐matched controls during the follow‐up period. AA, alopecia areata; AD, atopic dermatitis; CI, confidence interval; IR, incidence rate; NMSC, nonmelanoma skin cancer; PY, person‐years.

IRs were higher in the AD cohort compared with the non‐AD control cohort for malignancies, including NMSC (IR/100 000 PY, 107.5 [95% CI, 03.0–112.2] vs 89.0 [95% CI, 84.9–93.3]) and malignancies excluding NMSC (107.1 [95% CI, 102.5–111.8] vs 88.8 [95% CI, 84.7–93.1]; Figure 4a). IRs of these outcomes were higher in the psoriasis cohort compared with the nonpsoriasis cohort controls and lower in the AA and vitiligo cohort compared with the matched controls, albeit with overlapping 95% CIs. IRs for individual solid tumor malignancies assessed in this analysis, including breast, lung, gastric, pancreatic, and colorectal cancers, were comparable between disease cohorts and their matched controls (Figure 4b). IRs were higher in the AD cohort than in the non‐AD control cohort for lymphoma (IR/100 000 PY, 13.8 [95% CI, 12.2–15.6] vs 5.7 [95% CI, 4.7–6.8], respectively), cutaneous T‐cell lymphoma (1.6 [95% CI, 1.1–2.2] vs 0.1 [95% CI, 0.0–0.4], respectively), and leukemia (4.8 [95% CI, 3.9–5.9] vs 3.0 [95% CI, 2.2–3.8], respectively).

Similarly, IRs were higher in the psoriasis cohort compared with the nonpsoriasis control cohort for lymphoma (IR/100 000 PY, 50.6 [95% CI, 38.9–64.6] vs 17.2 [95% CI, 10.8–26.1], respectively) and cutaneous T‐cell lymphoma (12.6 [95% CI, 7.2–20.5] vs 0.8 [95% CI, 0.0–4.4], respectively) (Figure 4b). The IR for cutaneous T‐cell lymphoma was higher in the AA cohort than the non‐AA control cohort (IR/100 000 PY, 6.3 [95% CI, 1.7–16.2] vs 0.0 [95% CI, 0.0–5.8], respectively), with overlapping 95% CIs. IRs for lymphoma, cutaneous T‐cell lymphoma, and leukemia were comparable in the vitiligo and nonvitiligo control cohorts.

### 3.5 Incidence of infections

**FIGURE 5:**
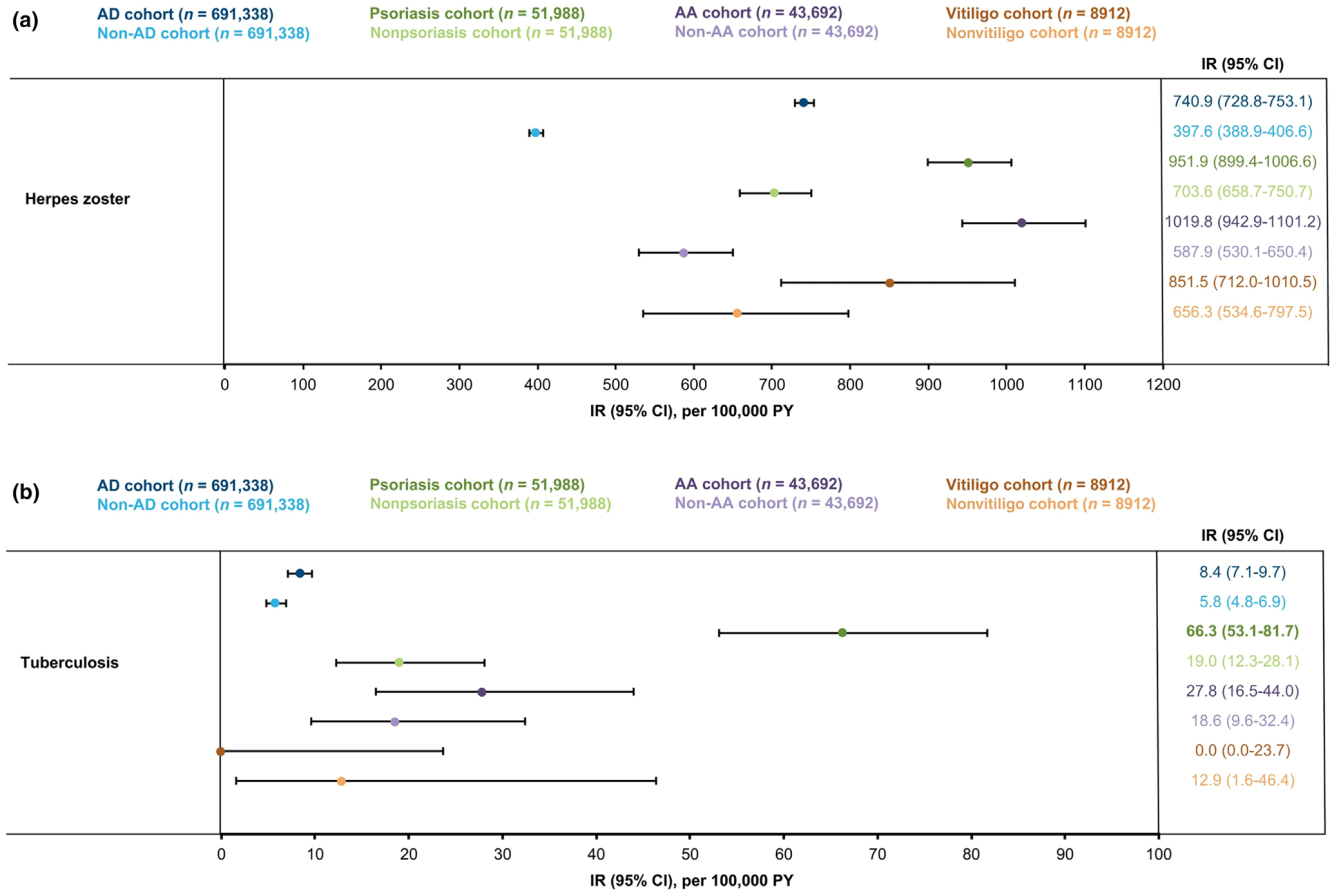
IRs of (a) herpes zoster infections and (b) tuberculosis in patients with AD, psoriasis, AA, and vitiligo compared with index month‐, age‐, and sex‐matched controls during the follow‐up period. AA, alopecia areata; AD, atopic dermatitis; CI, confidence interval; IR, incidence rate; PY, person‐years.

The IR of herpes zoster was higher in all disease cohorts compared with matched controls (AD: IR/100 000 PY, 740.9 [95% CI, 728.8–753.1] vs non‐AD: 397.6 [95% CI, 388.9–406.6]; psoriasis, 951.9 [95% CI, 899.4–1006.6] vs nonpsoriasis, 703.6 [95% CI, 658.7–750.7]; AA, 1019.8 [95% CI, 942.9–1101.2] vs non‐AA, 587.9 [95% CI, 530.1–650.4]) but with overlapping CIs in the vitiligo versus nonvitiligo control cohorts (851.5 [95% CI, 712.0–1010.5] vs 656.3 [95% CI, 534.6–797.5]; Figure 5a). Similarly, the IRs of tuberculosis were higher in the AD and psoriasis cohorts relative to their matched controls (AD: IR/100 000 PY, 8.4 [95% CI, 7.1–9.7] vs non‐AD: 5.8 [95% CI, 4.8–6.9]; psoriasis: 66.3 [95% CI, 53.1–81.7] vs 19.0 [95% CI, 12.3–28.1]; Figure 5b). The incidence of tuberculosis was comparable in the AA and vitiligo cohorts compared with their matched controls.

### 3.6 Incidence of comorbidities by age

IRs for major cardiovascular events, including ischemic heart diseases and stroke, malignancies, and infections (herpes zoster and tuberculosis), generally increased with age among all disease and respective control cohorts (Figures S2–S5). IRs were highest in the age 60 years and older subgroup and were lowest in the age 0 to 9 years and 10 to 19 years subgroups among all cohorts. The incidences of comorbidities were generally similar between disease cohorts and matched controls, with the following exceptions: the IR of all malignancies in the age 60 years and older subgroup was higher in the AD cohort versus the control cohort, and the IR of VTE events was higher among all age category subgroups in the AD cohort compared with matched controls. Furthermore, the IR for herpes zoster was also higher in all age category subgroups for all disease cohorts compared with matched controls; CIs were overlapping in some age category subgroups in the psoriasis, AA, and vitiligo matched cohorts (Figures S2–S5).

## 4 DISCUSSION

This retrospective cohort study aimed to assess the prevalence and incidence of comorbidities, including CVDs, malignancies, infections, dermatologic diseases, and other related conditions in patients from Japan with AD, psoriasis, AA, and vitiligo using data from the JMDC claims database. Allergic diseases (including allergic rhinitis, conjunctivitis, and asthma), dermatologic conditions, and infections were the most common comorbidities, and prevalence during the study baseline period was generally higher in patients with skin diseases compared with control cohorts matched by index month, age at index month, and sex. Furthermore, we found that the IRs of major cardiovascular events, VTE events, malignancies, and herpes zoster and tuberculosis infections were higher in patients with AD and psoriasis relative to the matched controls during the study follow‐up period; 95% CIs often overlapped for AA and vitiligo versus controls.

In this study, IRs of overall malignancies, including or excluding NMSC, were higher in the AD and psoriasis cohorts but comparable in the AA and vitiligo cohorts versus matched controls. Most notably, IRs were higher for lymphoma and cutaneous T‐cell lymphoma in AD cohorts (lymphoma: 13.8 [95% CI, 12.2–15.6] vs 5.7 [95% CI, 4.7–6.8] and cutaneous T‐cell lymphoma: 1.6 [95% CI, 1.1–2.2] vs 0.1 [95% CI, 0.0–0.4]) and psoriasis (lymphoma: 50.6 [95% CI, 38.9–64.6] vs 17.2 [95% CI, 10.8–26.1] and cutaneous T‐cell lymphoma: 12.6 [95% CI, 7.2–20.5] vs 0.8 [95% CI, 0.0–4.4]) versus matched controls. The IRs of other cancer types assessed in this study were similar in the disease and matched control cohorts, with widely overlapping CIs. Previous studies in other countries have also reported a higher incidence of lymphoma in patients with AD and psoriasis.^43^, ^44^ A UK‐based cohort study using The Health Improvement Network (THIN) database found no differences in the incidence of overall malignancies between patients with or without AD; however, AD was associated with an elevated risk of lymphoma, which increased with disease severity.^43^ Another population‐based matched cohort study of patients from England and Denmark found that the absolute rates of lymphoma were higher in patients with atopic eczema than age‐ and sex‐matched controls.^9^ Similarly, a US‐based cohort study reported a higher incidence of lymphoma in patients with psoriasis versus matched controls (IR per 1000 PY, 1.4 [95% CI, 1.3–1.5] vs 0.7 [95% CI, 0.6–0.8]).^44^ A systematic review and meta‐analysis of cancer risk in patients with AD reported high heterogeneity among studies,^45^ suggesting that interpretation of our results warrants careful evaluation. The increased risk of lymphoma reported in patients with AD may suggest that patients with AD are more likely to develop lymphoma; however, because the initial clinical presentation of AD and cutaneous T‐cell lymphoma can be similar, delayed differential diagnosis may also be a factor in this observation.^46^, ^47^, ^48^ In addition, some patients with AD may undergo skin biopsy related to their diagnosis, potentially leading to more lymphoma claims. Another contributing factor to the high IR of lymphoma observed in our study may include a large number of claims for thymus and activation‐regulated chemokine (TARC) tests in patients from Japan with AD and comorbid lymphoma. In a study using data derived from the JMDC database, the rate of TARC testing increased after it was covered by Japanese medical insurance.^49^

In our study, the incidences of major cardiovascular events and VTE events were higher in the AD and psoriasis cohorts but similar, with widely overlapping CIs, in the AA and vitiligo cohorts versus matched controls. In the AA cohort, IR for ischemic heart diseases that led to hospitalization was higher compared with matched controls. Previously published studies have reported a higher risk of CVD outcomes in patients with AD and psoriasis compared with individuals without these diseases,^1^, ^5^ although findings have been inconsistent in the literature. A population‐based cohort study in Taiwan reported a 1.33‐fold higher incidence of ischemic stroke in patients with AD compared with matched controls.^50^ In another study in the United Kingdom, the estimated IRs for stroke were similar between patients with or without atopic eczema; however, patients with severe AD had a 20% increased risk of stroke.^1^ A Danish population cohort also found higher event rates for ischemic stroke in patients with psoriasis versus a reference population, which increased with disease severity.^15^ Results from our study of patients in Japan underscore the need for physicians to consider the risk of these comorbidities when managing patients with dermatologic diseases.

The present analysis identified a high prevalence of infections among other comorbidities in patients with AD, psoriasis, AA, and vitiligo compared with matched control cohorts, and most notably for viral and fungal infections among all disease cohorts. These results are consistent with previous studies using data from the JMDC, which found that comorbidities such as viral and fungal infections were more prevalent in patients with AD and AA compared with matched controls.^39^, ^51^ In addition, in our study, IRs of herpes zoster were higher among all disease cohorts, with overlapping CIs in the vitiligo cohort compared with matched controls; whereas, IRs for tuberculosis were higher in the AD, psoriasis, and AA cohorts, with overlapping CIs in the AA and non‐AA matched cohorts. These results are consistent with observations in a population‐based cohort study in the United Kingdom, which also reported increased risk of infections, including herpes zoster and herpes simplex, in patients with AD.^10^ In AD, increased risk for infections may be due to the immunosuppressive effects of systemic therapy in some patients with severe disease in whom these therapies are recommended.^52^ Furthermore, systemic treatments such as corticosteroids or cyclosporine A may be associated with increased risk for infections, including tuberculosis, as well as for CVDs and malignancies.^31^, ^37^, ^53^, ^54^, ^55^, ^56^, ^57^, ^58^ Patients in this study may have used these treatments, which may, in part, explain the higher observed IRs for these comorbidities.

Our analysis found that the prevalence of AD was higher in the psoriasis, AA, and vitiligo cohorts compared with their respective control cohorts. These findings support results from previously published studies of patients in Japan, including a study of patients from the same JMDC database, which also showed a high prevalence of comorbid AD in patients with AA.^39^ In general, there may be bias toward detection of comorbid dermatologic conditions in the disease cohorts due to their frequent visits to the dermatologist compared with matched cohorts. In our analysis, the finding that AD was common in patients with psoriasis may also be biased due to physicians prescribing AD medications such as tacrolimus for psoriasis treatment, thereby increasing AD insurance claims. Vitiligo is common in patients treated with immune checkpoint inhibitors for malignancies^59^; however, the effect of these therapies was not analyzed in this study.

The present analysis also showed that IRs of comorbidities, including major cardiovascular events, malignancies, and infections, were higher in older versus younger patients among all disease cohorts. IRs for many of the comorbidities evaluated were similar between dermatologic disease cohorts and matched controls within age categories, with some notable exceptions. In the AD cohort, the IRs of all malignancies were higher in patients aged 60 years and older and IRs of VTE events were higher in those aged 40 years and older, versus matched controls. In all disease cohorts, IRs for herpes zoster were higher in each age category compared with matched controls, albeit with overlapping CIs in the psoriasis, AA, and vitiligo cohorts. Studies conducted in several countries have also reported that the incidences of cardiovascular events and malignancies in patients with dermatologic diseases tend to increase with age.^43^, ^60^, ^61^ A UK cohort–based study found that the incidence of malignancies was higher in adult versus pediatric patients with AD and, more importantly, that pediatric and adult patients with moderate or severe AD had higher rates of malignancies compared with their respective non‐AD–matched controls.^43^

Unique sets of immunologic mechanisms underlie AD, psoriasis, AA, and vitiligo pathophysiology; AD and psoriasis are predominantly Th2‐ and Th17‐mediated, respectively, while AA and vitiligo are predominantly Th1‐mediated.^29^, ^30^, ^62^ The increased prevalence of dermatologic diseases observed in patients with AD, psoriasis, AA, and vitiligo compared with controls may indicate some overlap in the T‐cell–mediated inflammatory pathways. Patients with psoriasis and comorbid AD have been described^63^, ^64^; however, studies characterizing this patient population are limited.

Multiple studies have reported on the psychosocial burden of AD, AA, vitiligo, and psoriasis, with deleterious consequences for patients' quality of life.^18^, ^19^, ^20^, ^21^, ^22^ In this analysis, psychosocial conditions, including sleep disorders, symptoms of anxiety, and depression, were found to be among the common comorbidities observed among the study cohorts; however, their prevalence was comparable to individuals in the matched control cohorts. Sleep disorders, symptoms of anxiety, and depression are often assessed using patient‐reported outcome tools, and these results may suggest that patients from Japan do not view their dermatologic conditions as having impact on their mental health; however, further studies are needed to examine these relationships.

The JMDC database has been used as a data source to analyze disease burden and treatment patterns of patients from Japan in other studies.^65^ Patient cohorts described in this analysis generally had similar demographics (i.e., age and ratio of male‐to‐female participants), when compared with previously described cohorts for AD, psoriasis, and AA from the same JMDC database.^39^, ^51^, ^66^ Similar to this analysis, asthma, allergic rhinitis, and AD were identified as some of the common comorbidities in patients with AA in a previous descriptive study using data collected from the JMDC database.^39^

A limitation of this study is that it was not designed for inferential statistical analysis; therefore, the prevalence and incidence data are only descriptive, and direct comparisons between patient cohorts cannot be made. Several other potential limitations are inherent to the JMDC data source, a payer‐based database of claims from health insurance societies that cover employees and their dependent family members. JMDC includes few participants aged 65 to 75 years, and none of the participants in our study were aged 75 years or older. Health insurance societies that provide the claims data to JMDC may not always be representative of health insurance societies in Japan and they could exhibit bias by location and/or industry. In addition, not all outcomes were validated among disease populations. The accuracy of AD, psoriasis, AA, and vitiligo diagnoses in this database has not been extensively characterized. However, an increasing number of studies support the validity of the data from the JMDC, particularly for inpatient diagnoses.^65^ Another limitation of this study is that IRs were not determined for all outcomes that were found to be prevalent in the disease cohorts. This study analyzed IRs of events that may be deemed as adverse events of special interest, especially in patients with dermatologic diseases, including AD, psoriasis, and AA, who may have a higher risk of developing these events (e.g., cardiovascular events, malignancies, and serious infections) when undergoing treatment.^40^, ^41^, ^42^ One strength of the current study is the assessment of these events by age categories, which provides insight for different risk factors that may be considered during clinical management.

In conclusion, the findings from this study show that the prevalence and incidence of certain comorbidities were higher among individuals from Japan with dermatologic diseases, particularly those with AD and psoriasis, compared with individuals without dermatological diseases. This observational retrospective cohort study using patient data from Japan found that allergic or atopic diseases (including allergic rhinitis, conjunctivitis, and asthma), dermatologic conditions, and infections were the most frequently occurring comorbidities, and their prevalence was higher in patients with AD, psoriasis, AA, and vitiligo compared with controls matched by age, sex, and index month. In addition, patients with these dermatologic diseases exhibited generally higher incidences of major cardiovascular events, VTE events, malignancies (lymphoma and cutaneous T‐cell lymphoma), and infections (herpes zoster and tuberculosis) compared with matched controls; however, 95% CIs often overlapped for AA and vitiligo cohorts versus matched controls. Further research is needed to examine these associations and potential causal relationships between these comorbidities and AD, psoriasis, AA, and vitiligo. Awareness of the risks of comorbidities in patients with skin diseases in Japan may inform clinician decision‐making around disease management. Current guidelines from The Japanese Dermatological Association highlight the importance of the management of comorbidities of skin diseases, including allergic diseases, bacterial, fungal, and viral infections.^67^

## FUNDING INFORMATION

This study, including the collection, analysis, and interpretation of data and writing of the report, was sponsored by Pfizer Japan Inc., Tokyo, Japan.

## CONFLICT OF INTEREST STATEMENT

YM, MC, TH, and KN are employees of Pfizer Japan Inc. NS and SH are employees of Pfizer R&D Japan G.K. MC, TH, KN, SH, and NS are shareholders of Pfizer Inc. SI has received grants, consulting fees and/or speaker's fees from Pfizer Inc., AbbVie, Amgen Inc., Boehringer Ingelheim, Bristol Myers Squibb, Daiichi‐Sankyo, Eisai, Eli Lilly and Company, GlaxoSmithKline, Janssen, Kyowa Kirin, LEO Pharma, Maruho, Novartis, Sun Pharma, Taiho Pharmaceutical, Mitsubishi Tanabe, Torii Pharmaceutical, Toyo Pharmaceutical, Seiyaku Kasei, and UCB Japan.

## ETHICS STATEMENT

Approval of the research protocol by an institutional review board: N/A.

Informed consent: N/A.

Registry and the registration number of the study/trial: N/A.

Animal studies: N/A.

## Supporting information


Data S1.
