# Supplementary material for: Prevalence and incidence of comorbidities in patients with atopic dermatitis, psoriasis, alopecia areata, and vitiligo using a Japanese claims database
Source: J Dermatol. 2025 Feb 7;52(5):841–54. doi: 10.1111/1346-8138.17643 (PMC12056285; doi:10.1111/1346-8138.17643)

**Supplementary Material**

**Prevalence and incidence of comorbidities in patients with atopic dermatitis, psoriasis, alopecia areata, and vitiligo using a Japanese claims database**

Yue Ma^1,a^, Motohiko Chachin^1,a^, Tomohiro Hirose^1,*^, Kouki Nakamura^1^, Nanzhi Shi^2^, Shintaro Hiro^2^, Shinichi Imafuku^3^

^1^Pfizer Japan Inc., Tokyo, Japan; ^2^Pfizer R&D Japan, Tokyo, Japan; ^3^Department of Dermatology, Fukuoka University, Fukuoka, Japan

^a^These authors contributed equally and are co-lead authors of this manuscript.

*Corresponding author: Tomohiro Hirose

Specialty Care Medical Affairs,

Pfizer Japan Inc.

Shinjuku Bunka Quint Bldg.

3-22-7, Yoyogi,

Shibuya-ku, Tokyo

151-8589 Japan

Email: [tomohiro.hirose@pfizer.com](mailto:tomohiro.hirose@pfizer.com)

**Supplementary Table 1** Patient comorbidities and ICD-10 codes.

| **Conditions** | **ICD-10 code** |
| --- | --- |
| **Allergic diseases**^†^ | |
| Asthma | J45, J46 |
| Allergic rhinitis | J30 |
| Food allergy | T78.0, T78.1 |
| Conjunctivitis | B30, H10, H11 |
| **Infections**^†^ | |
| Bacterial infection | A00, A01, A03, A04, A05, A15-A19, A20-A28, A30-A49, A50-A64, A65-A69, A70-A74, A75-A79 |
| Viral infection | A08, A80-A89, A90-A99, B00-B09, B15-B19, B20-B24, B25-B34 |
| Fungal infection | B35-B49 |
| Herpes simplex | B000, B001, B002, B003, B004, B005, B007, B008, B009 |
| Herpes zoster | B02, B02.0, B02.1, B02.2, B02.3, B02.7, B02.8, B02.9 |
| Tuberculosis | A15-19 |
| **Mental health disorders**^†^ | |
| Anxiety disorder | F40-F45, F48 |
| Depression | F32, F33 |
| Sleep disorders | F51, G47 |
| Attention-deficit hyperactivity disorder | F90 |
| **Respiratory diseases**^†^ |  |
| Chronic obstructive pulmonary disease | J42–J44 |
| Chronic rhinosinusitis | J32 |
| Nasal polyps | J33 |
| **Malignancies** | |
| All malignancies including NMSC | C00-C97 excluding C76-C80, D00-D09 |
| All malignancies excluding NMSC | C00-C97 excluding C44, C76-C80, D00-D09 |
| Breast cancer | C50, D050, D051, D057, D059 |
| Lung cancer | C33-, C34-, D021, D022 |
| Gastric cancer | C16-D002 |
| Pancreatic cancer | C25 |
| Colorectal cancer | C18-, C19-, C20, D010, D011, D012 |
| Lymphoma | C81-C86, C88 |
| Cutaneous T-cell lymphoma | C84 |
| Leukemia | C91-95 |
| **Cardiovascular Diseases**^†^ | |
| Stroke | I60-64 |
| Hypertension | I10-I15 |
| Peripheral arterial disease | I70.2, I70.3-I70.8, I70.9 |
| MACE^‡^ | I20-25, I60-64 |
| Ischemic heart disease | I20-25 |
| VTE^§^ | I80, I82, O22, O87, O88, I26 |
| DVT | I80, I82, O22, O87, O88 |
| PE | I26 |
| **Metabolic disorders**^†^ | |
| Diabetes mellitus | E10-E14 |
| **Dermatosis** | |
| Acne**^§^** | L70 |
| Eczema herpeticum (Kaposi's varicelliform eruption)^†^ | B000 |
| AD | L20 |
| Psoriasis | L40 |
| AA | L63 |
| Vitiligo | L80 |
| **Autoimmune diseases**^†^ |  |
| Inflammatory bowel disease (along with ulcerative  colitis and Crohn’s disease) | K50–K52 |
| Lupus erythematosus | L93, M32 |
| Rheumatoid arthritis | M06, M05 [excluding M06.1, M06.4, M06.0 (RS3PE; Injury name master code: 8844120)] |
| Psoriatic arthritis | M07.0, M07.1, M07.2, M07.3, L40.5, M09.0 |
| Ankylosing spondylitis and non-infectious uveitis | M45, M08.1, H20 |
| Underwent organ transplantation | Z94, Y83.0, T86 |
| Celiac disease | K900 |
| Graves’ disease | E050 |
| Hashimoto thyroiditis | E060 |
| Sjogren’s syndrome | M350 |
| **Other diseases**^†^ | |
| Bone fracture | S42, S49.7, S52.5, S52.1-4/6-9, S59.7, S62, S69.7, S72.0-2, S72.3-9, S79.7, S82.0-4/7/9, S89.7, S82.5-6/8, S92.4-5, S92.0-3/6-9, S99.7 |
| Dyslipidemia | E78 |

Abbreviations: AA, alopecia areata; AD, atopic dermatitis; DVT, deep vein thrombosis; ICD, International Classification of Diseases 10th Revision; IR, incidence rate; MACE, major cardiovascular events; NMSC, nonmelanoma skin cancer; PE, pulmonary embolism; VTE, venous thromboembolism.

^†^At least 1 of the definitive diagnoses defined by the ICD-10 code

^‡^For analyses of IRs of major cardiovascular diseases, patients were excluded if any of the following ICD-10 codes were detected within 52 weeks during baseline period: ICD-10: I20-25 (any of ischemic heart disease diagnosis codes: acute coronary syndrome, and coronary artery disease), ICD-10: I60-64 (any stroke diagnosis code).

^§^For analysis of the incidence rate of VTE events, patients were excluded if any of the following ICD-10 codes were detected within 52 weeks during baseline period: ICD-10: I26, I260, I269, I80, I800, I801, I802, I803, I808, I809, I821, I822, I823, I828, I829, O082, O223, O871, O882 (any of VTE diagnosis code).

**Supplementary Figure 1.** Study period for patients with **(a)** new diagnosis and **(b)** prevalent diagnosis^a^


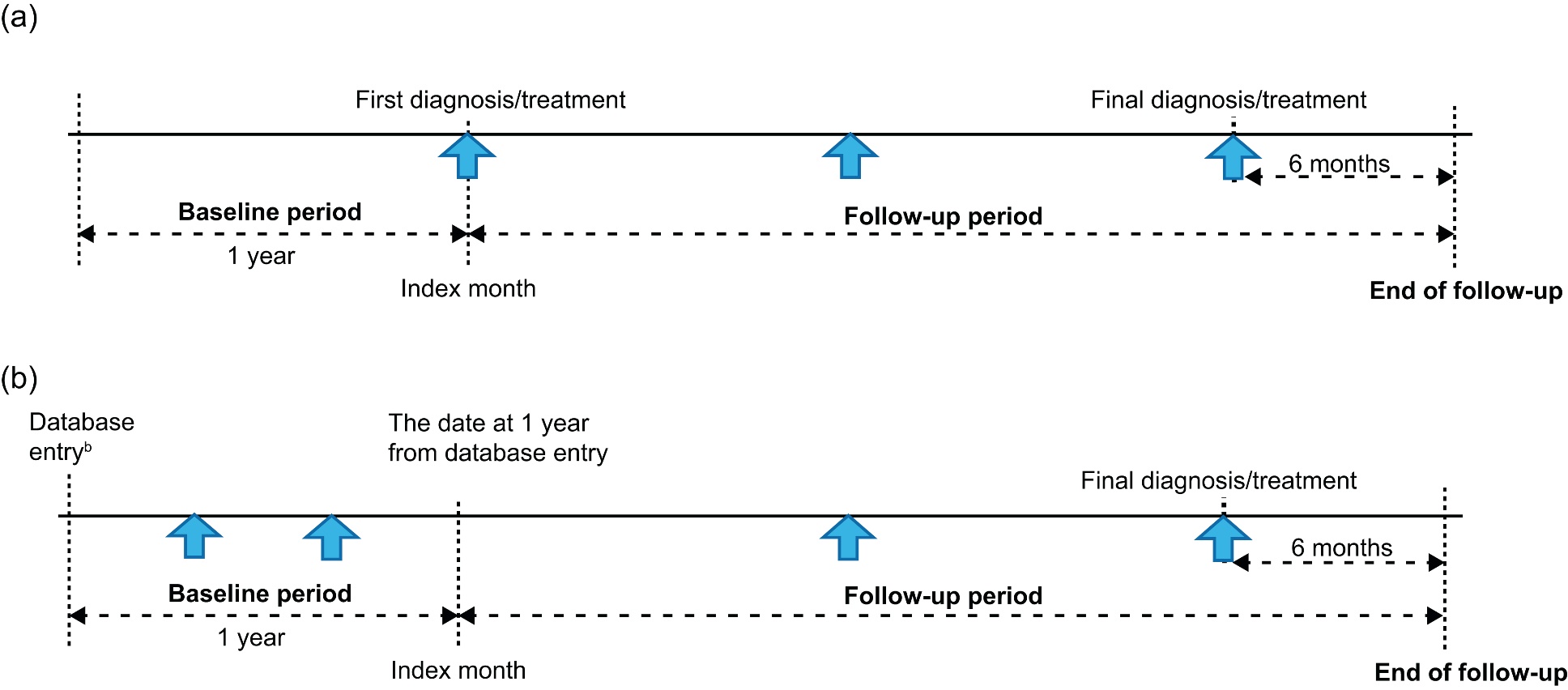


The baseline period, from June 2013 to December 2020, was defined as a 1-year interval prior to and including the index month. This period was used to obtain baseline information.

The index month was defined as the first record of the diagnosis or treatment of the four target dermatologic diseases – atopic dermatitis, psoriasis, alopecia areata, and vitiligo – after the baseline period. If the first diagnosis month was within the first year of a patient’s date of database entry, the index month was defined as the twelfth month after database entry and the year preceding the index month was defined as the baseline period.

For each initiation of an exposure of interest, the follow-up started from the first day of the next month after the index month. The follow-up period ended at the earlier date of either the loss of enrollment in insurance policy or 6 months after the final diagnosis/treatment.

^a^Baseline and follow-up periods were determined for patients in the JMDC database who had a previous diagnosis of one of the four dermatologic diseases (i.e., patients with prevalent diagnosis) or new diagnosis at database entry.

^b^If the date of database entry was prior to June 1, 2013, then June 1, 2013, was defined as the start date of the baseline period.

**Supplementary Figure 2**. IRs of comorbidities by age categories in index month-, age-, and sex-matched AD cohorts and controls. Abbreviations: AD, atopic dermatitis; CI, confidence interval; IR, incidence rate; NMSC, nonmelanoma skin cancer; PY, person-years.


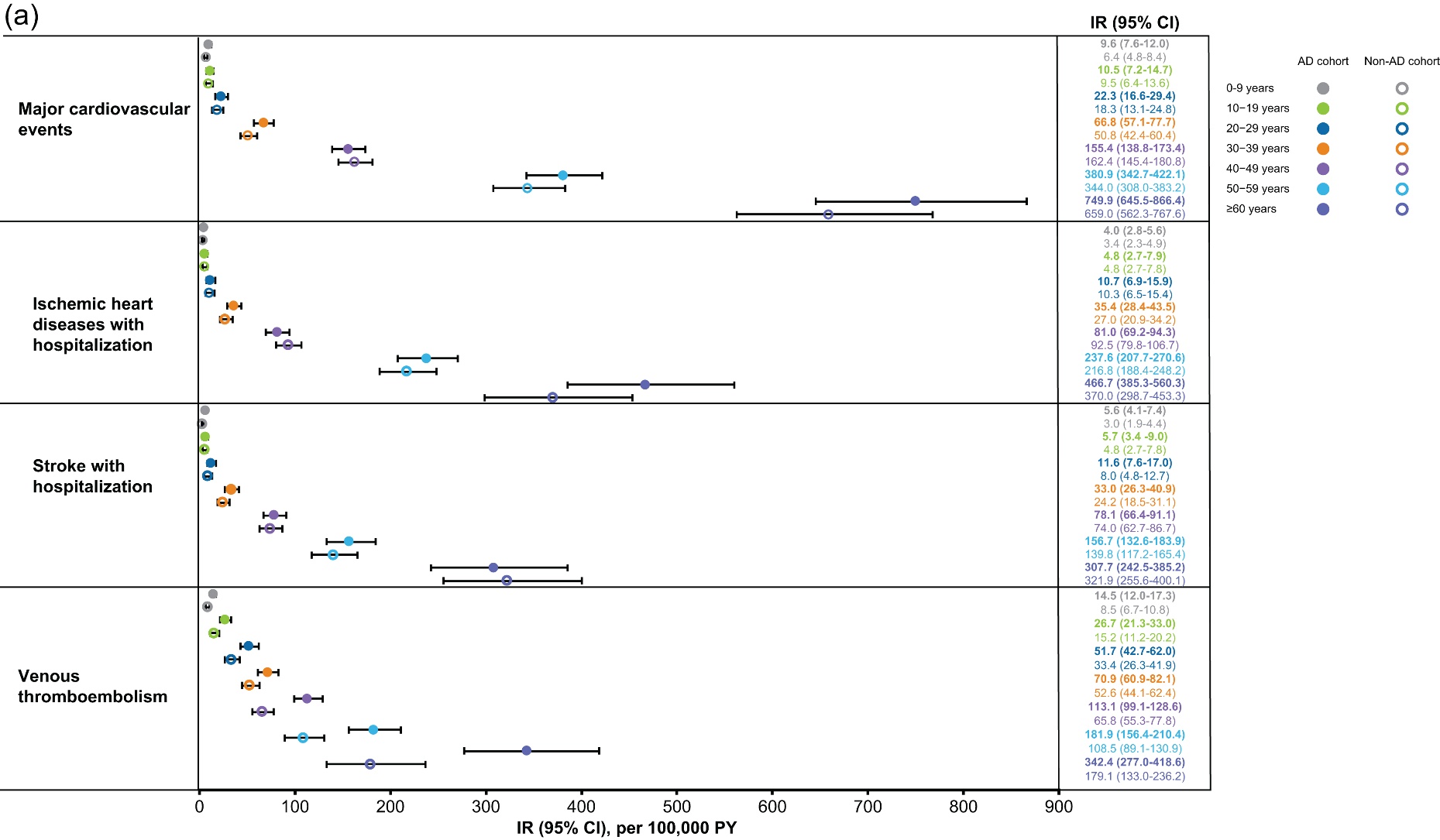


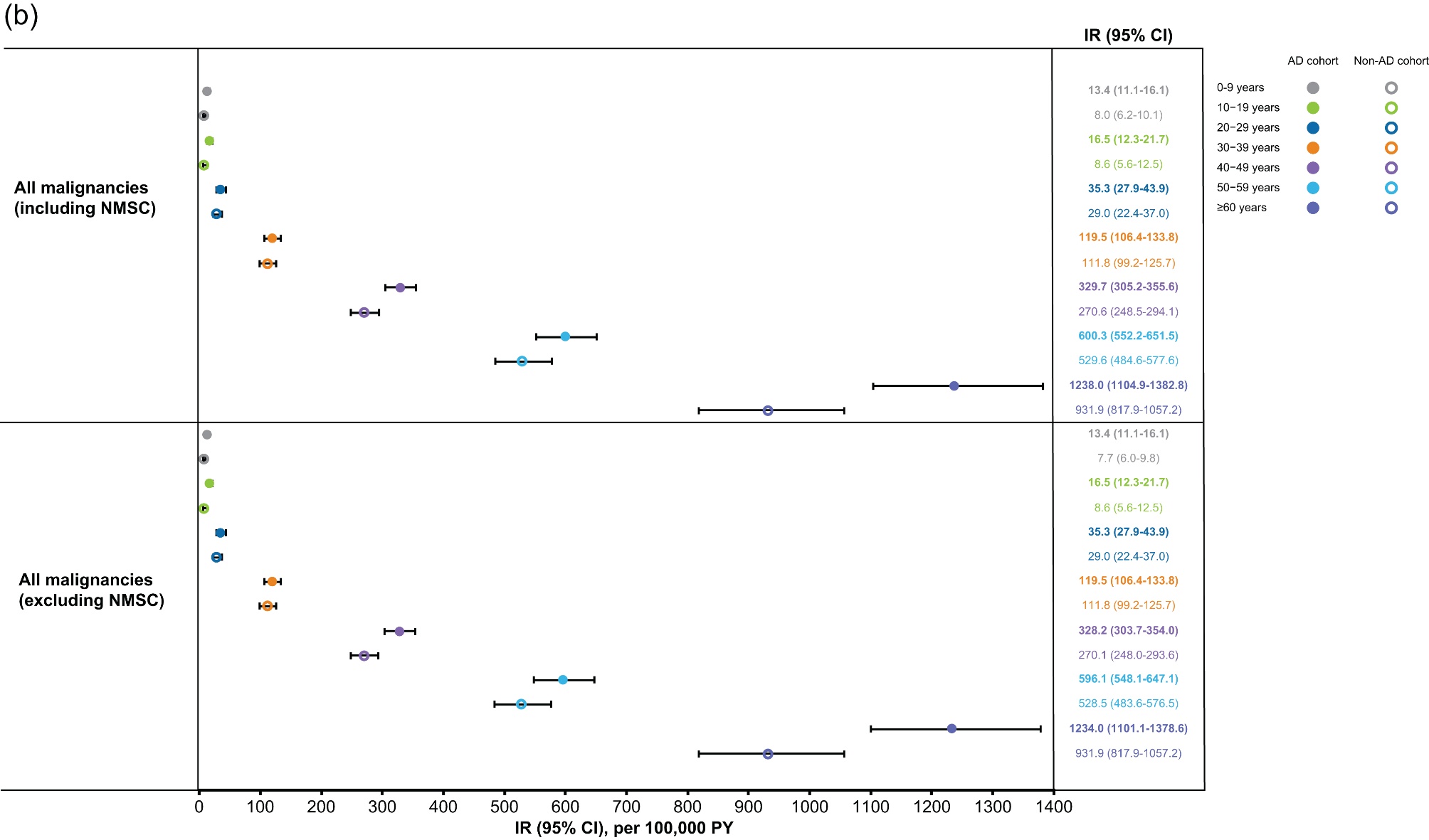


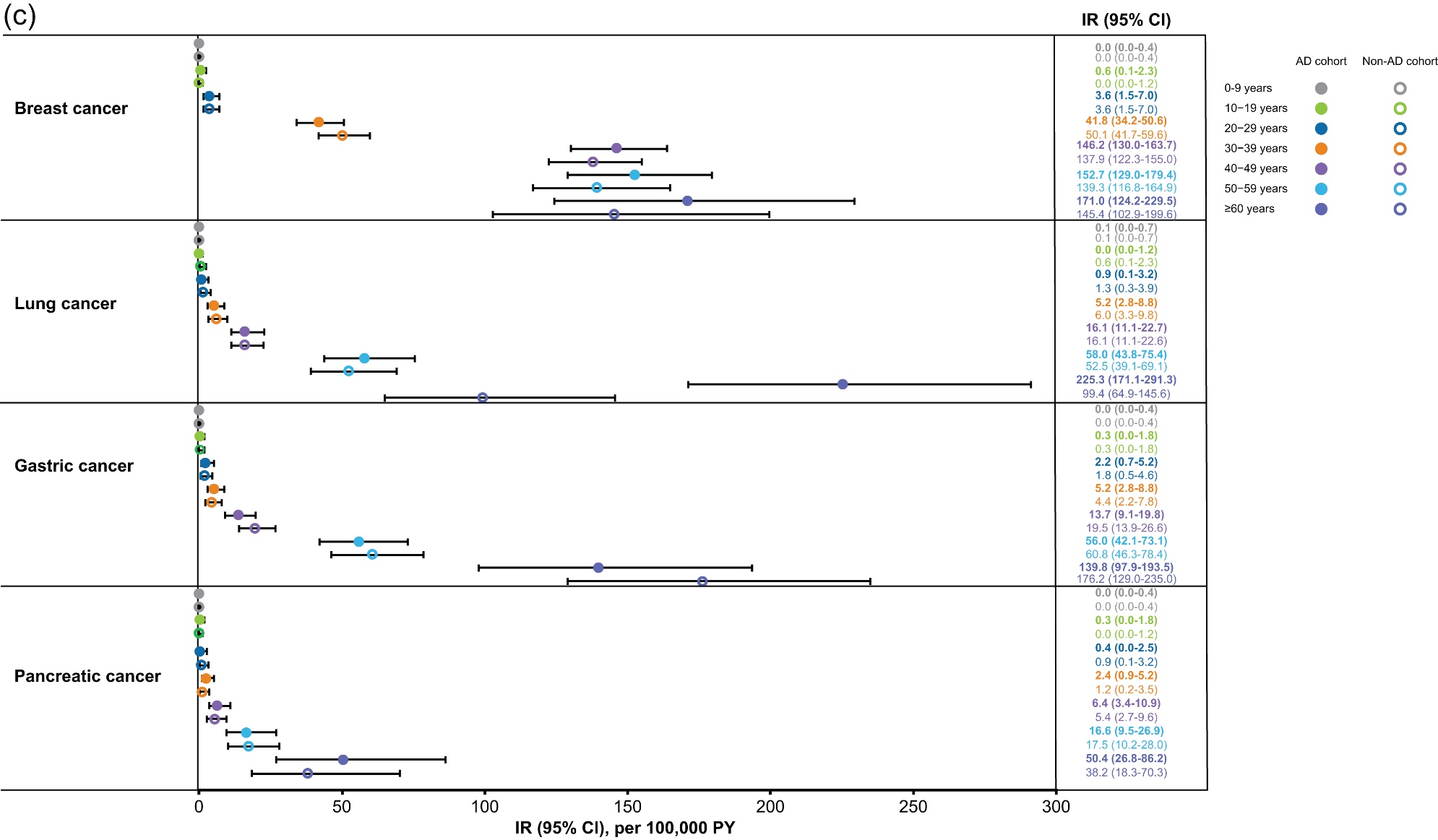


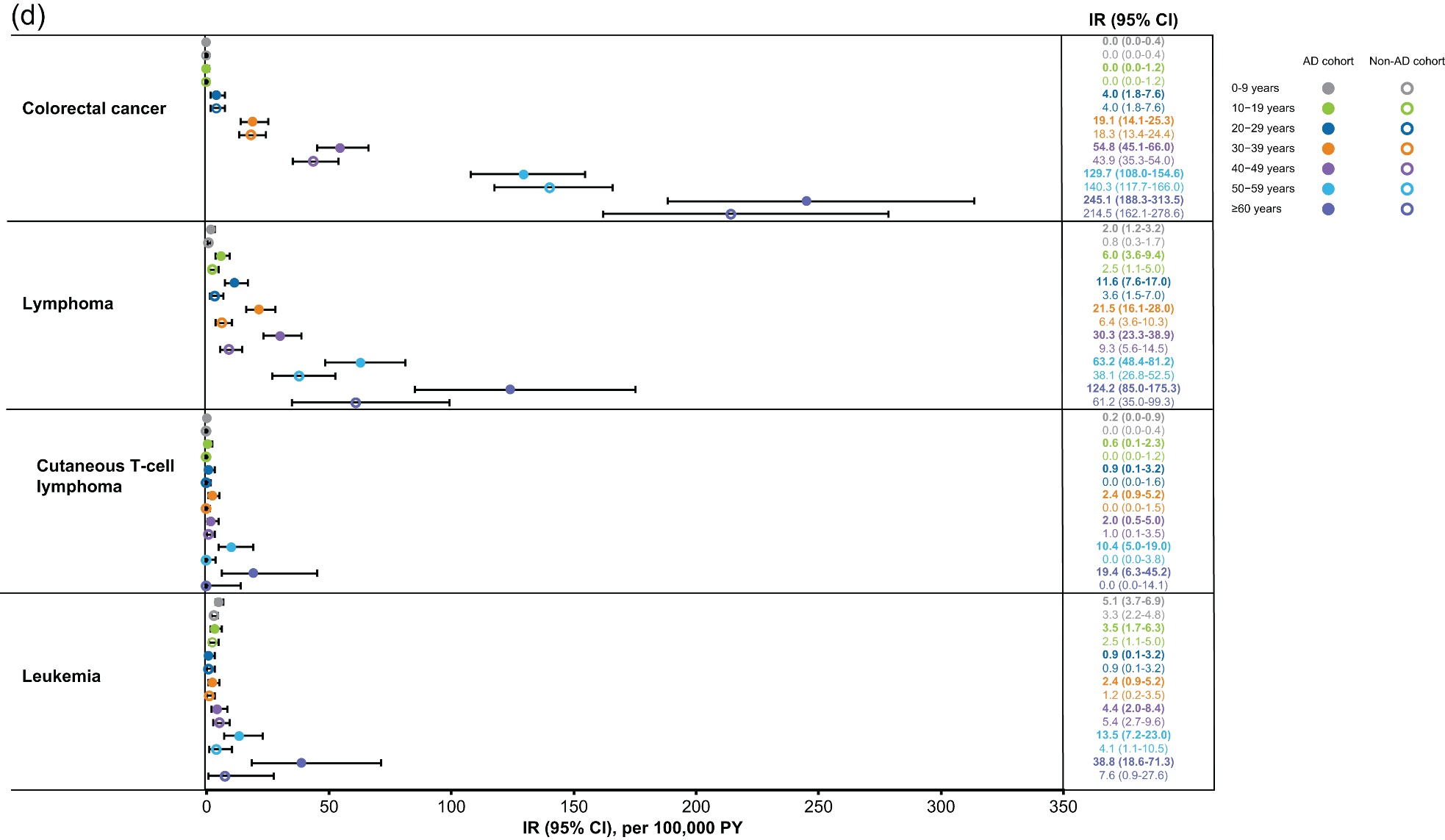


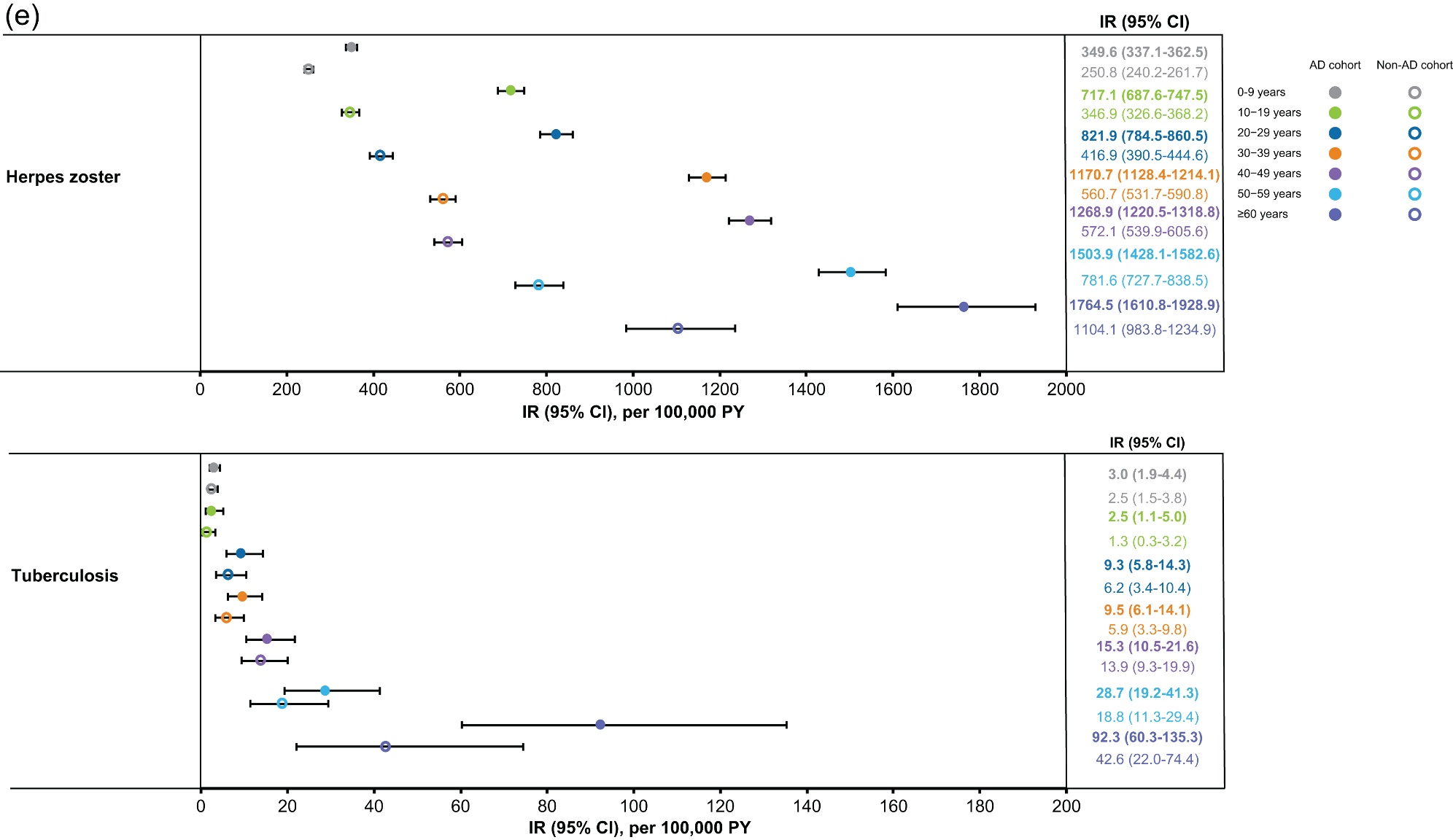


**Supplementary Figure 3**. IRs of comorbidities by age categories in index month-, age-, and sex-matched psoriasis cohorts and controls. Abbreviations: CI, confidence interval; IR, incidence rate; NMSC, nonmelanoma skin cancer; PY, person-years.


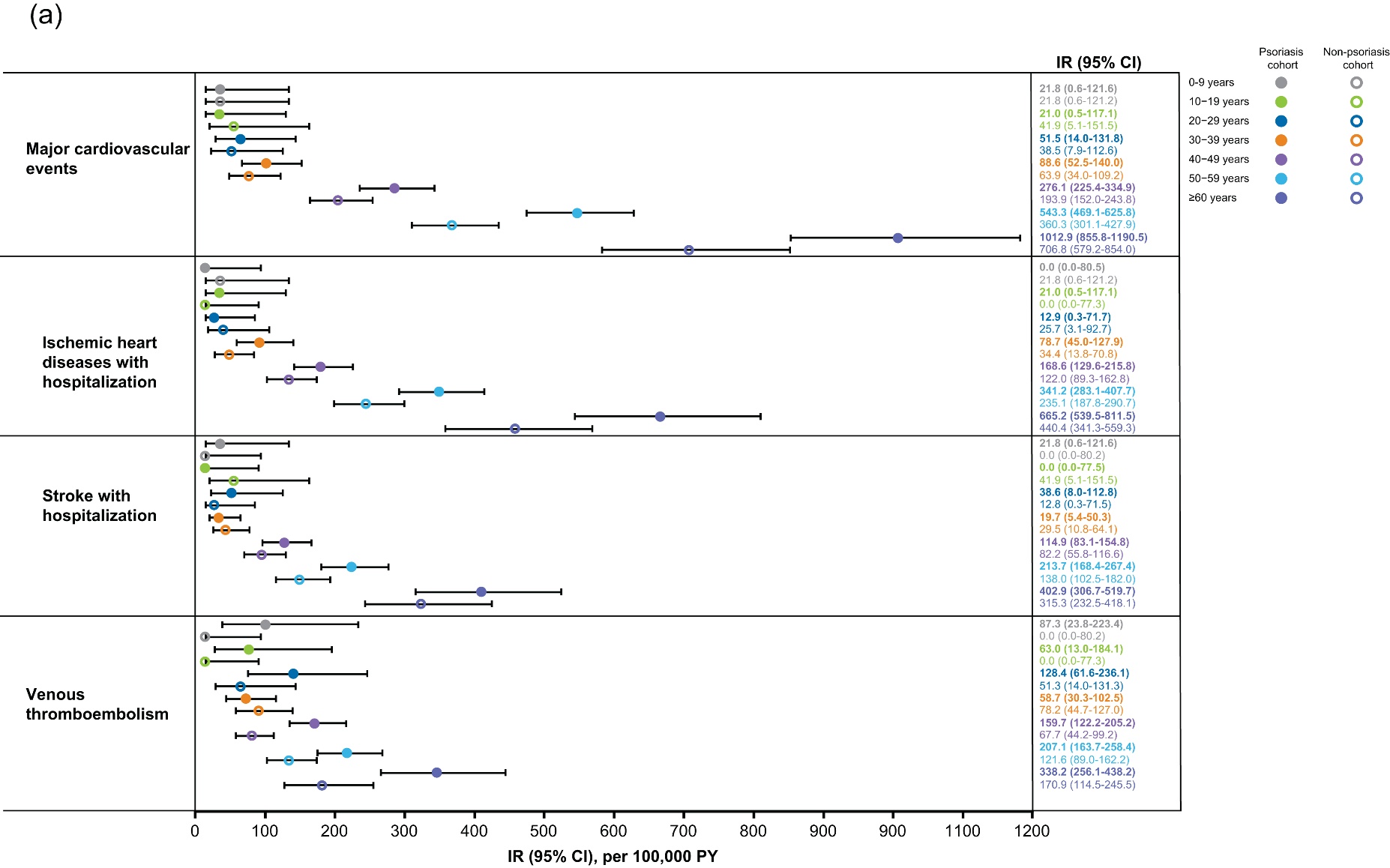


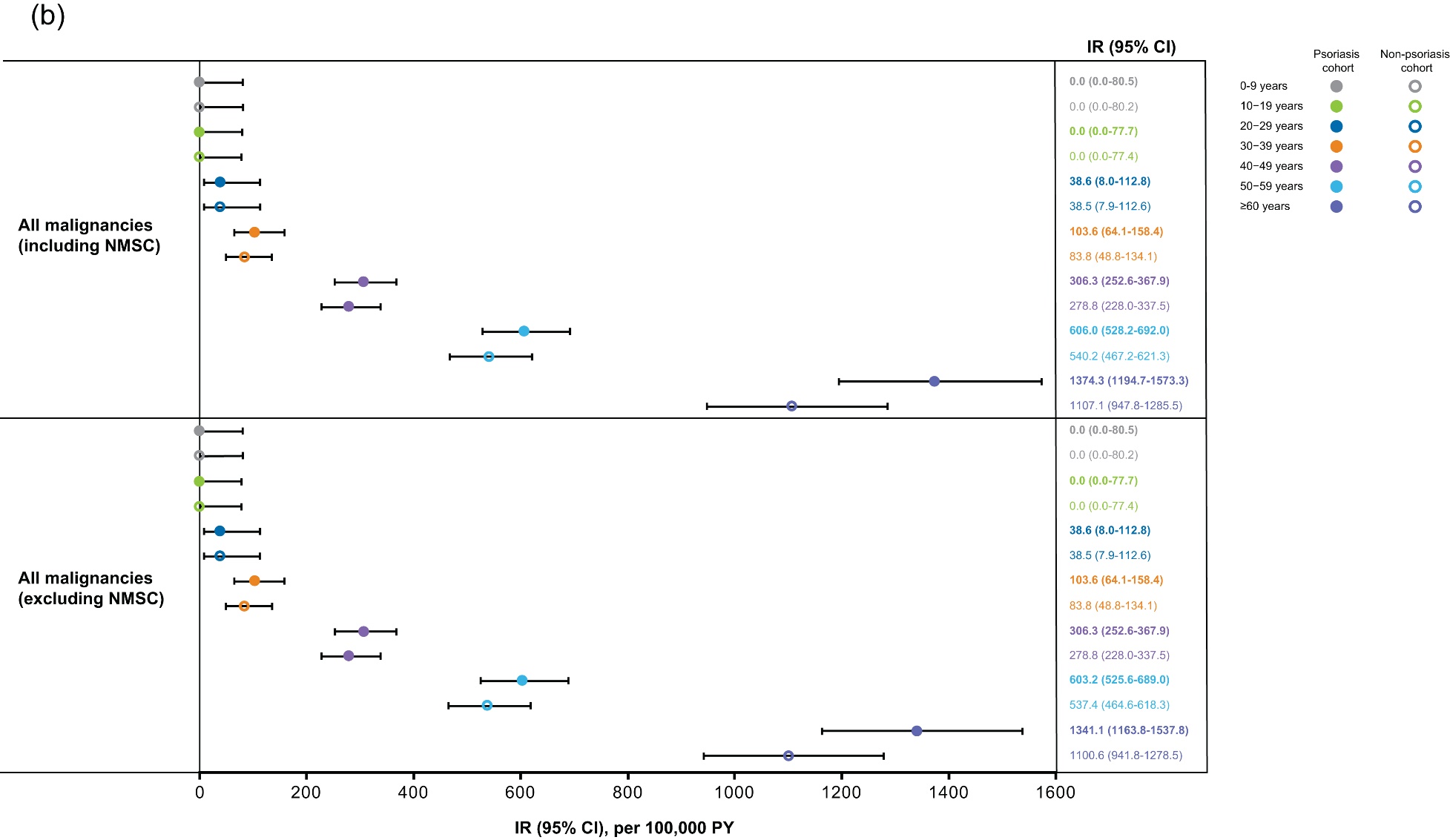


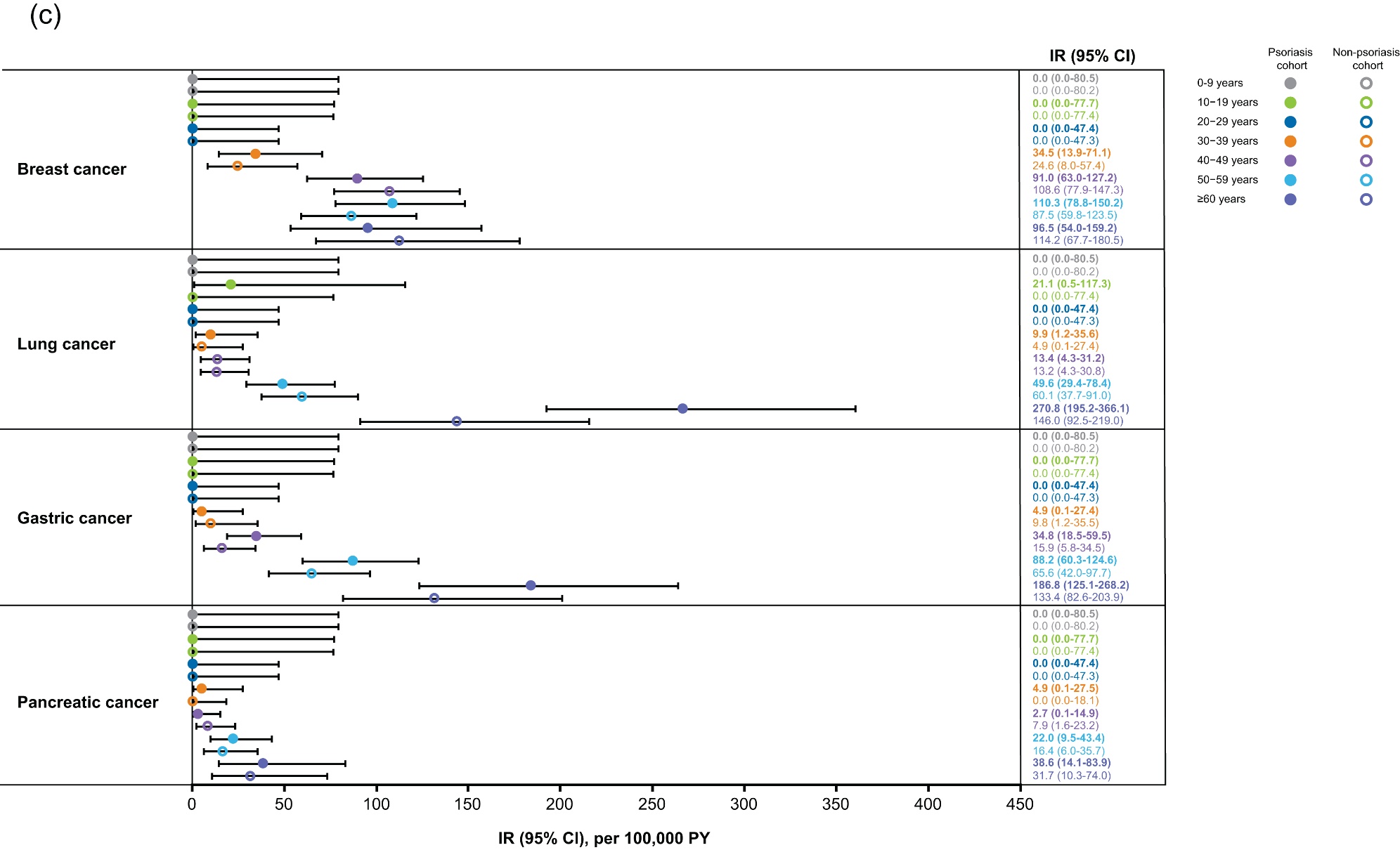


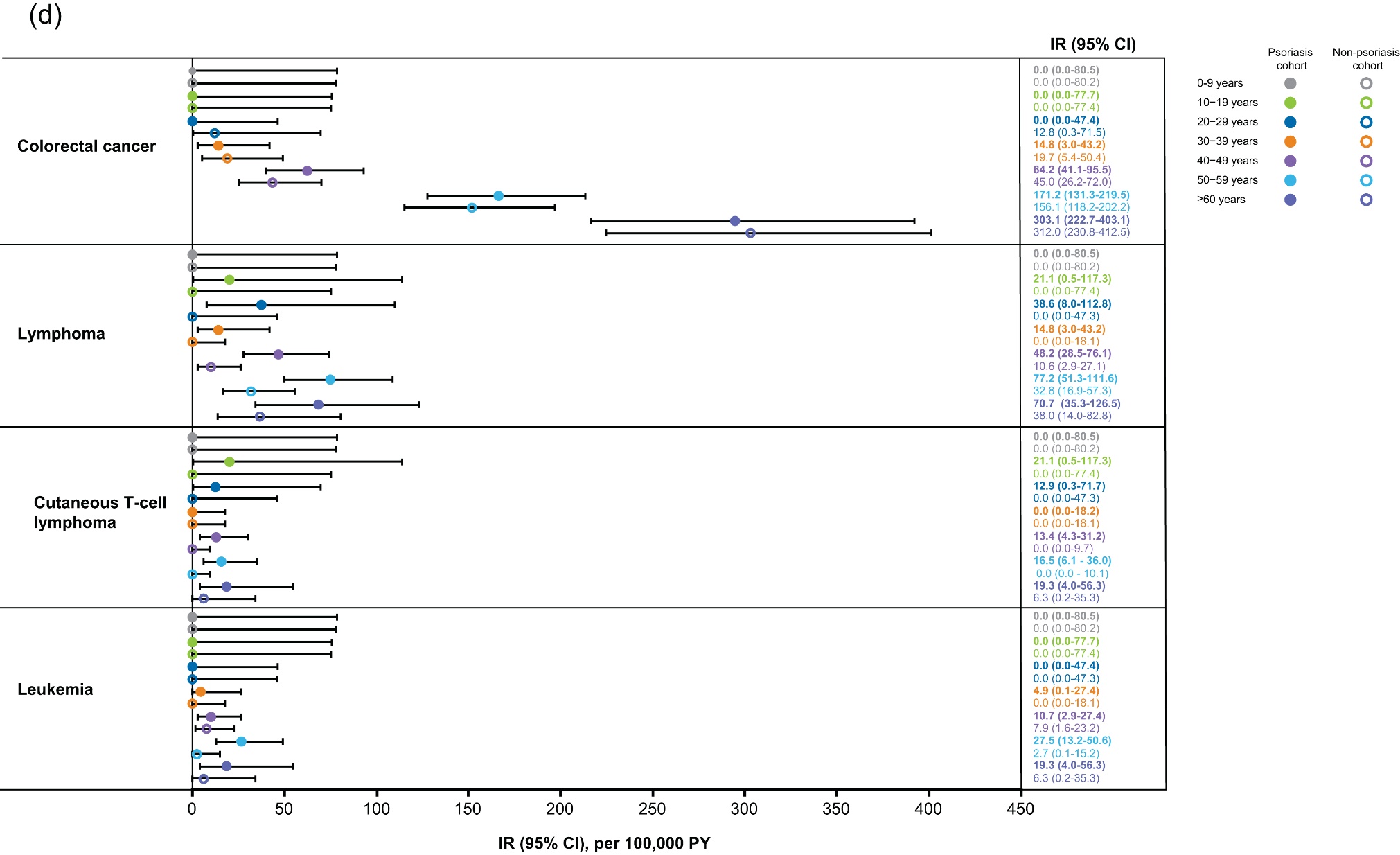


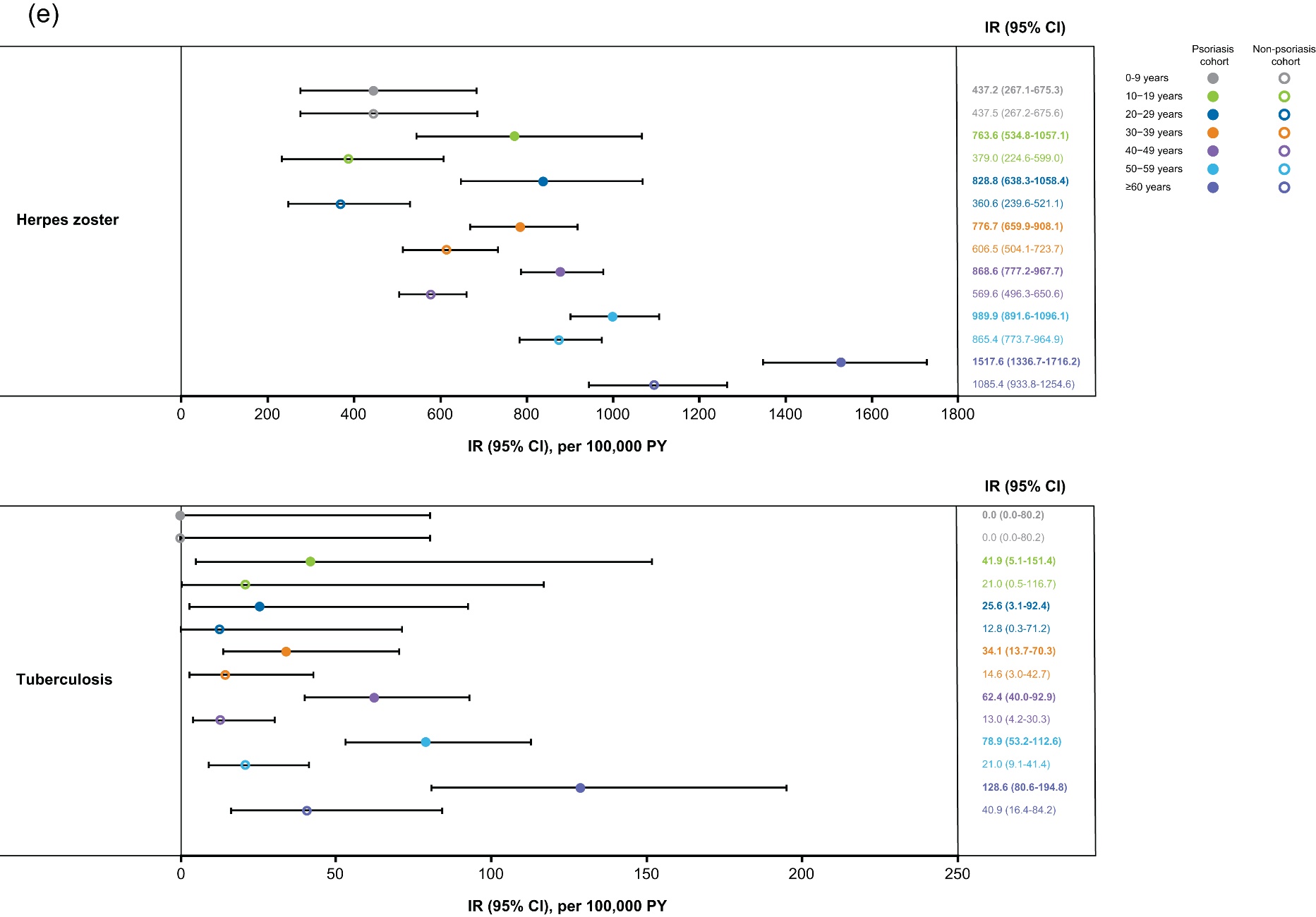


**Supplementary Figure 4**. IRs of comorbidities by age categories in index month-, age-, and sex-matched AA cohorts and controls. Abbreviations: AA, alopecia areata; CI, confidence interval; IR, incidence rate; NMSC, nonmelanoma skin cancer; PY, person-years.


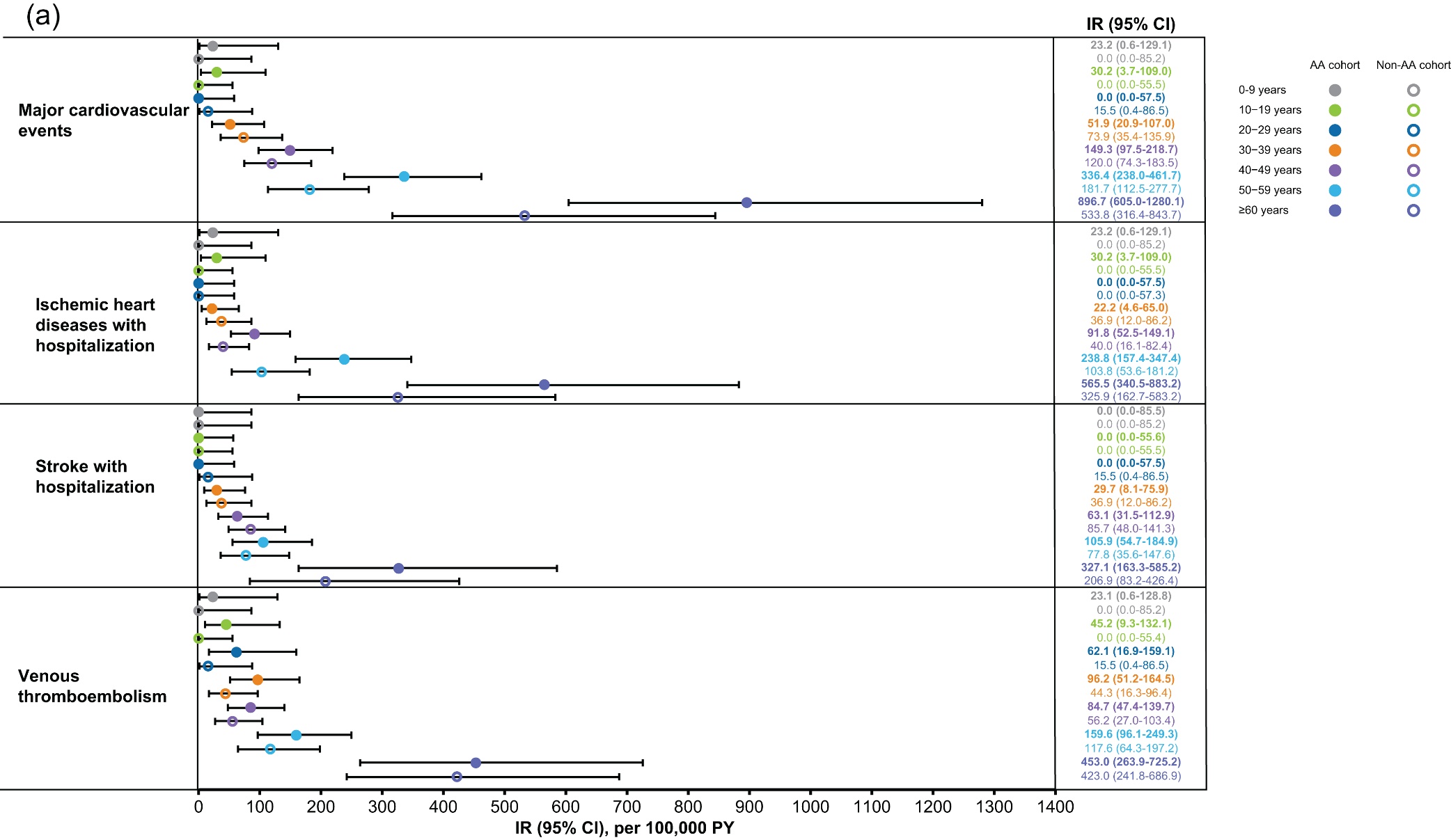


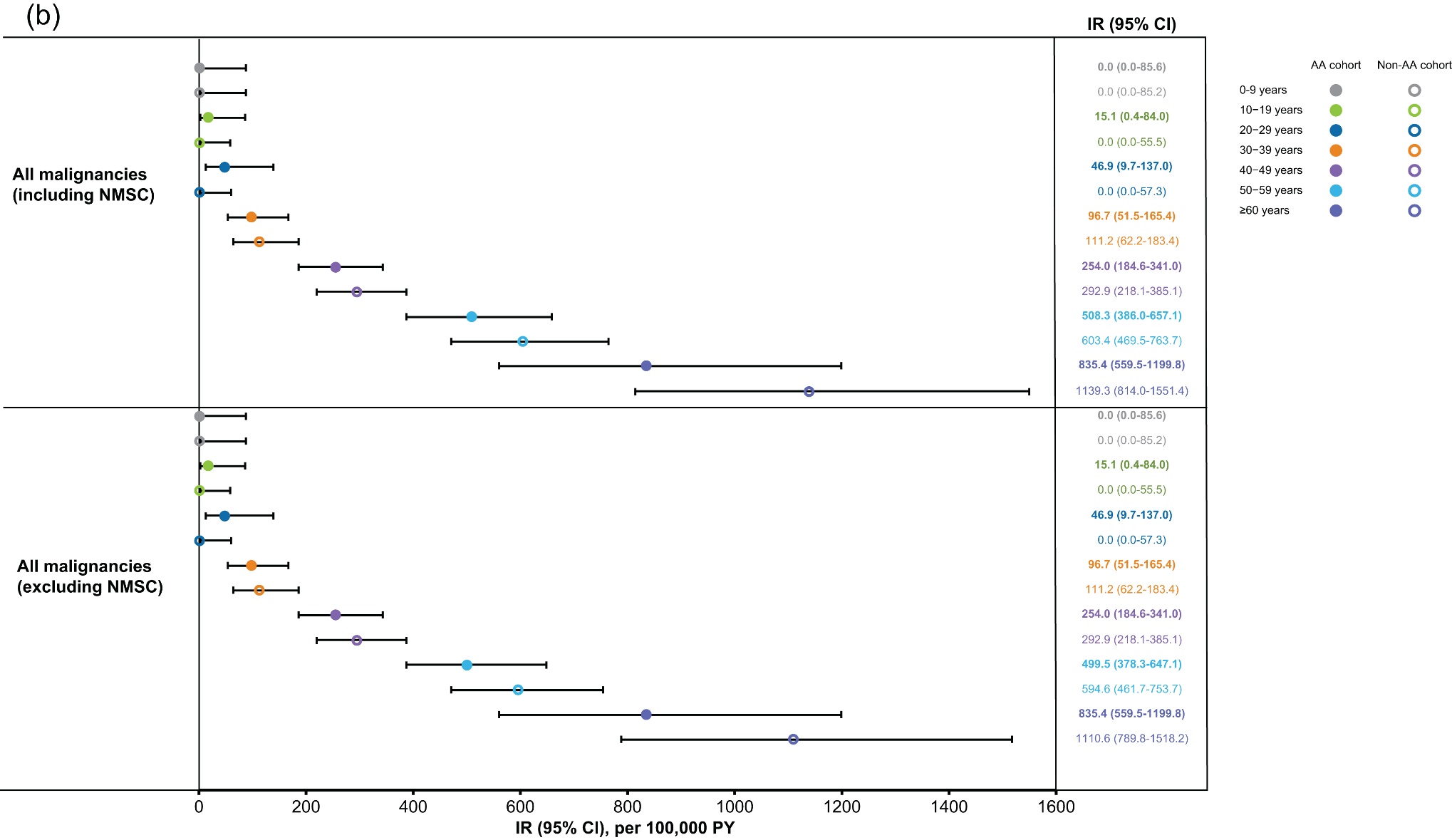


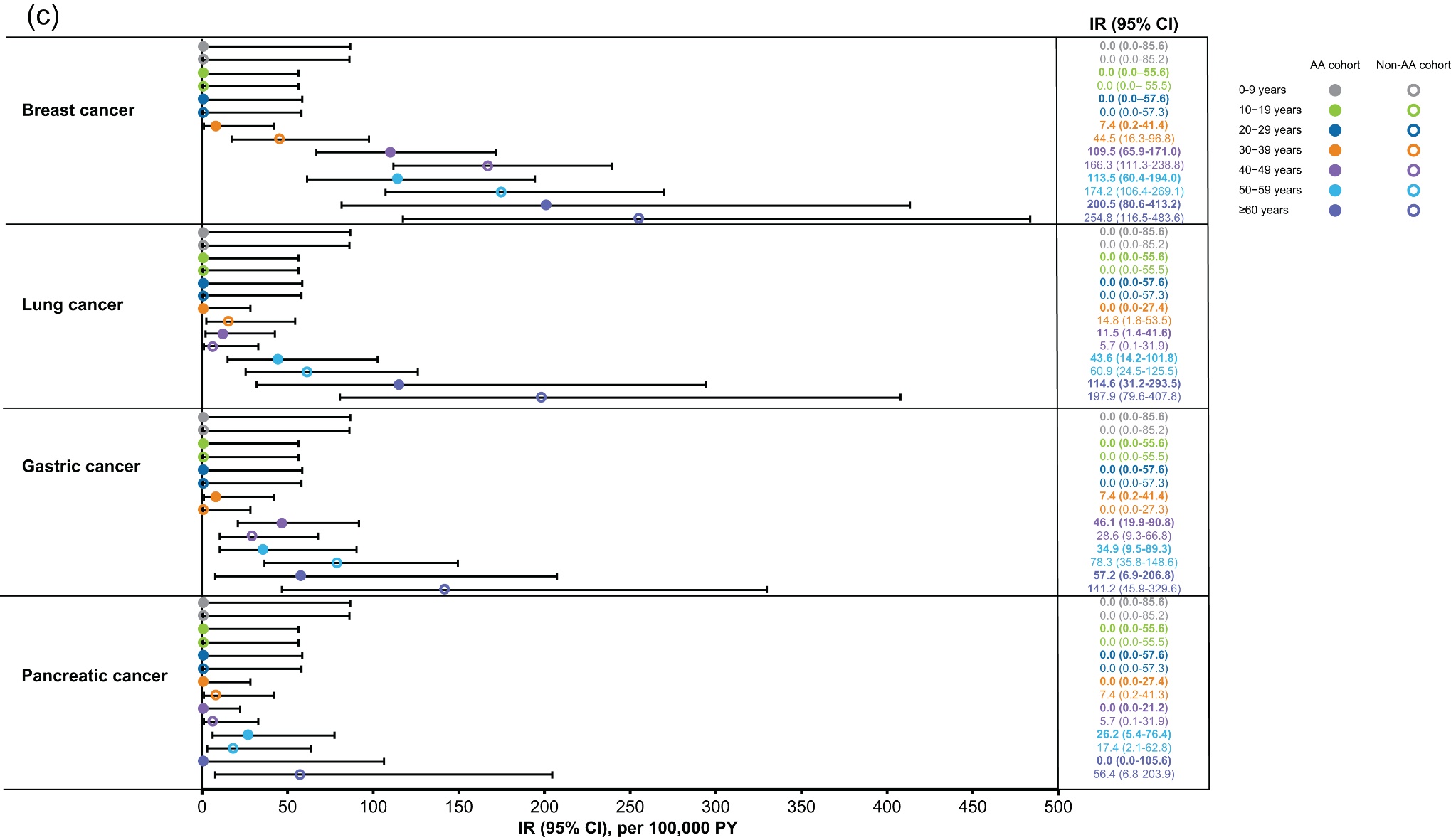


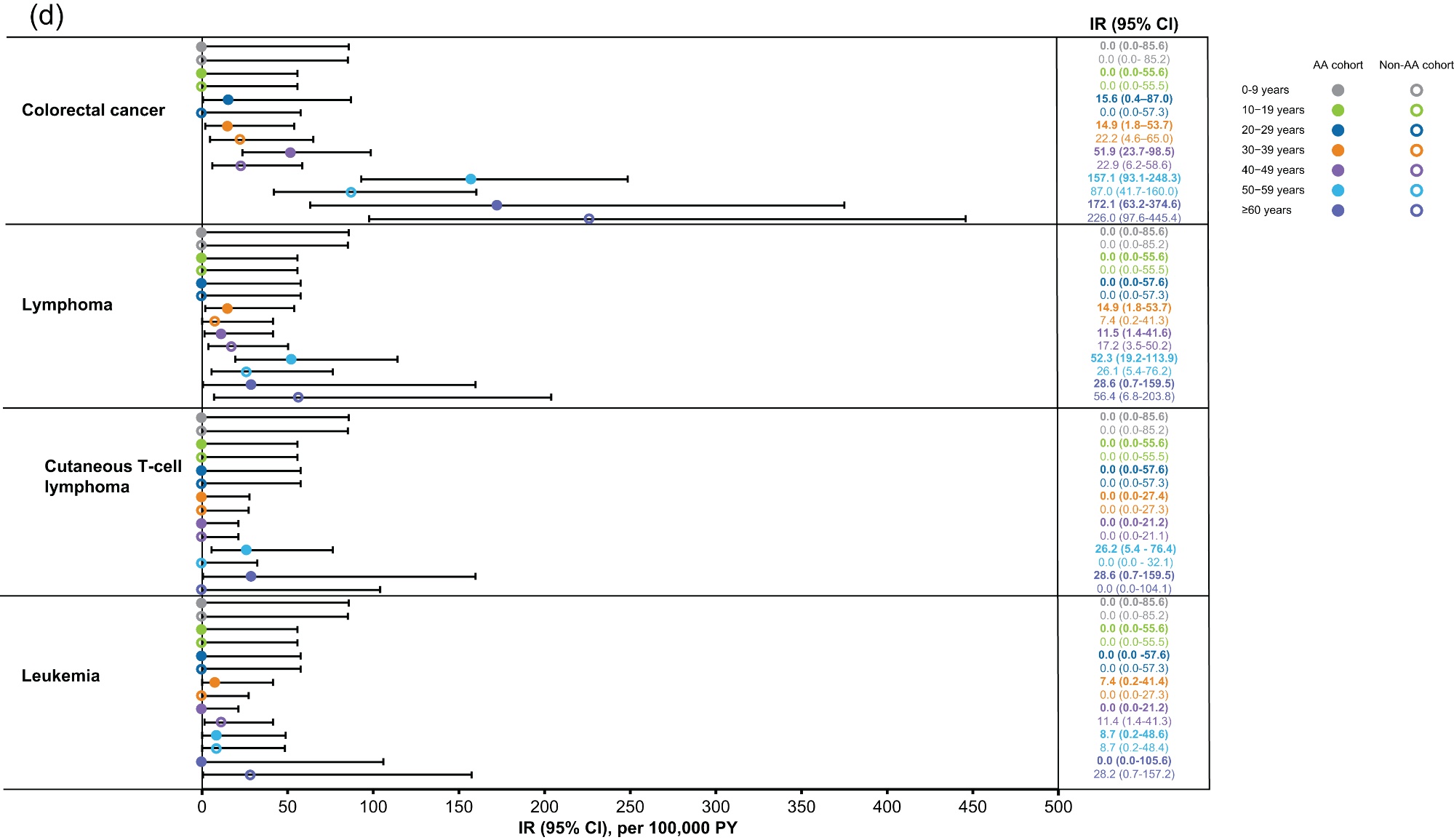


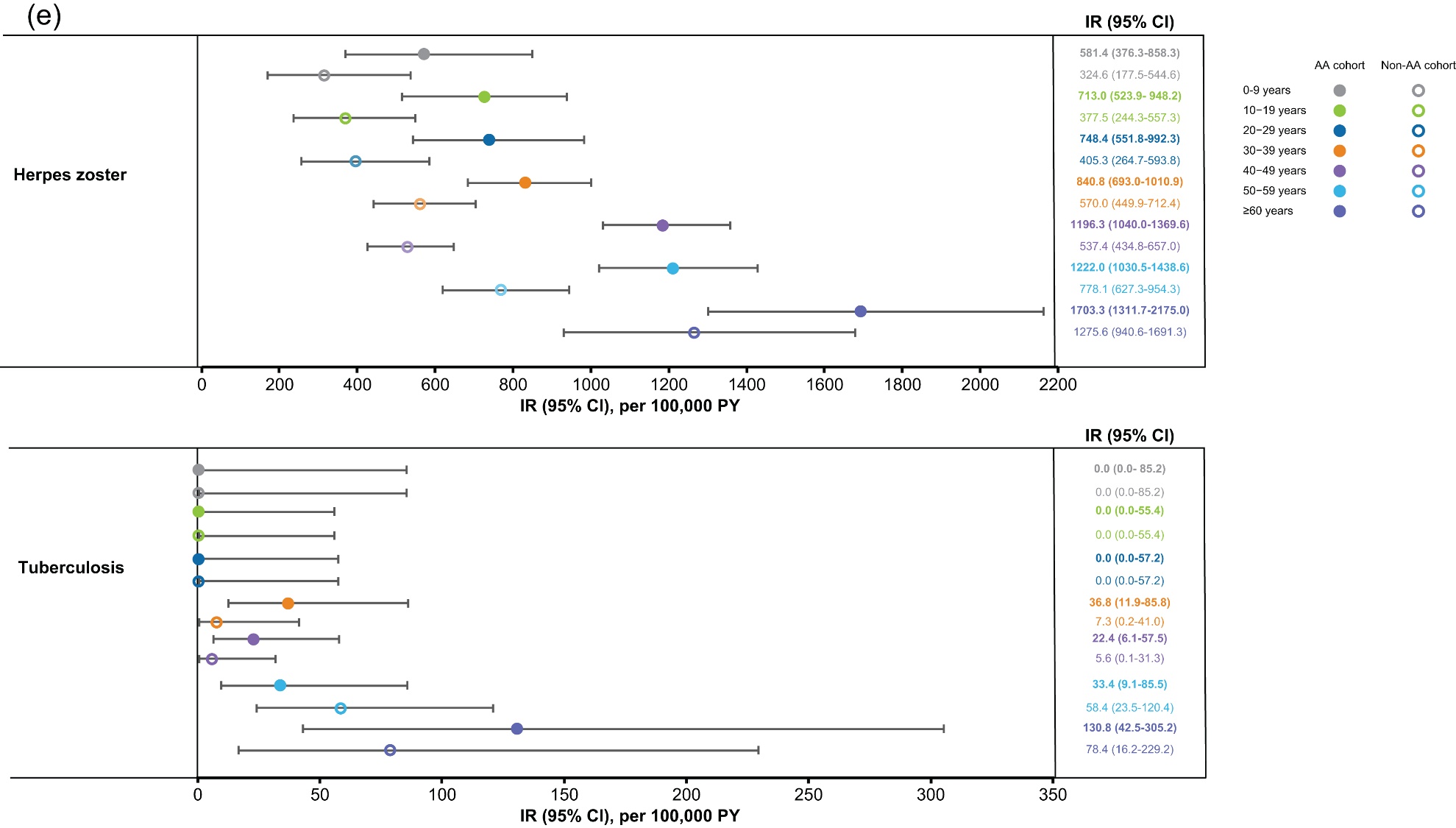


**Supplementary Figure 5.** IRs of comorbidities by age categories in index month-, age-, and sex-matched vitiligo cohorts and controls. Abbreviations: CI, confidence interval; IR, incidence rate; NMSC, nonmelanoma skin cancer; PY, person-years.


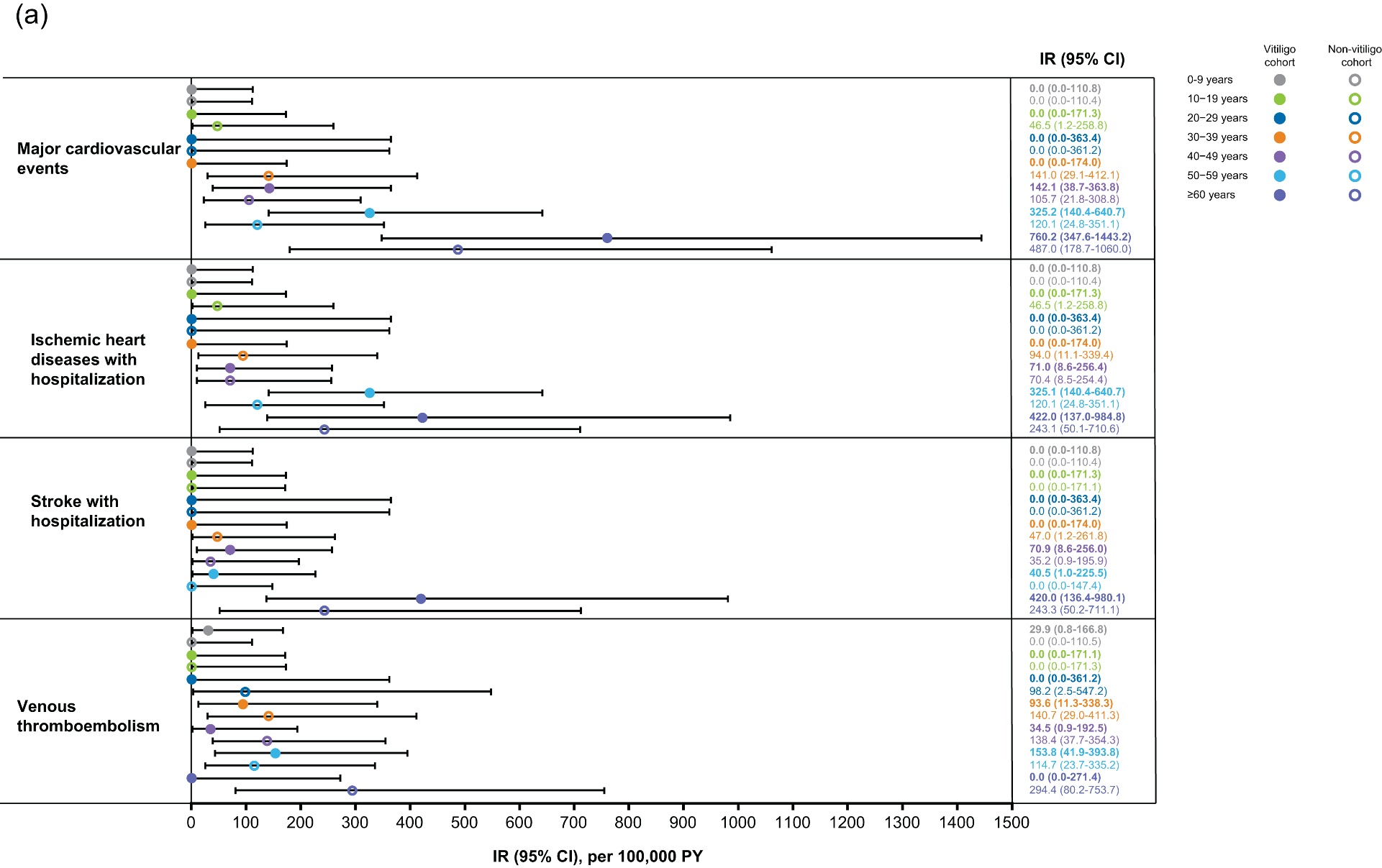


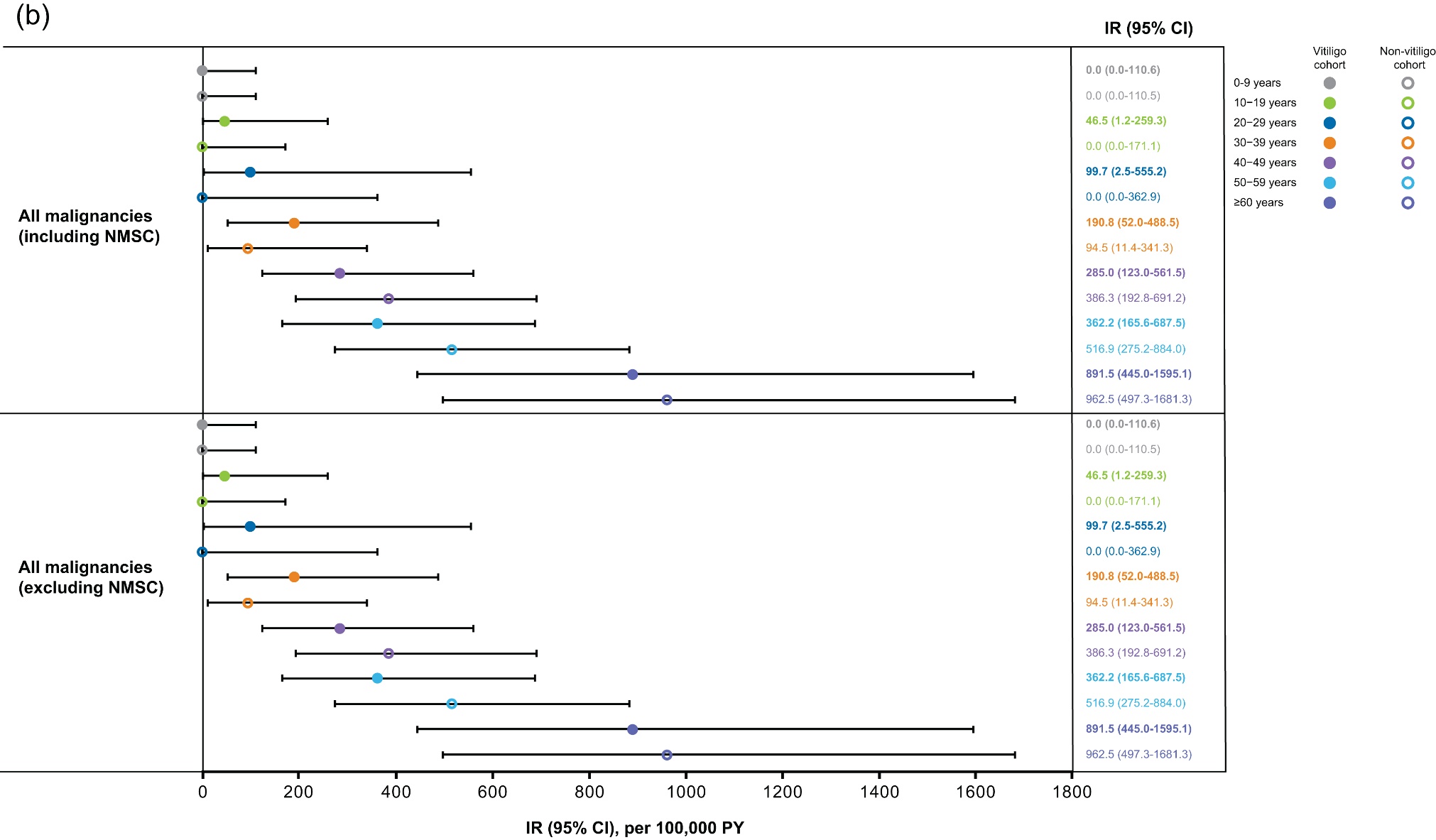


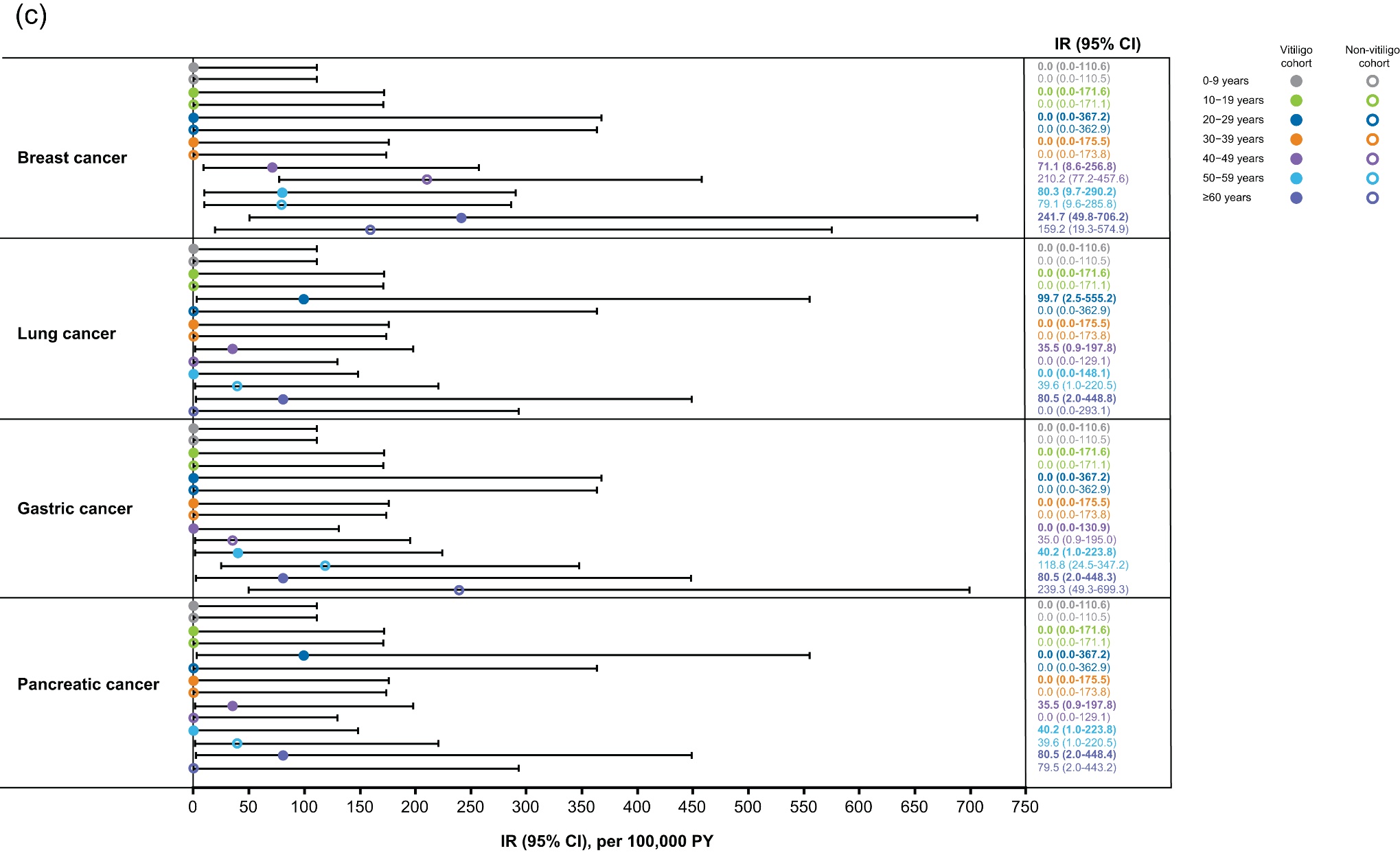


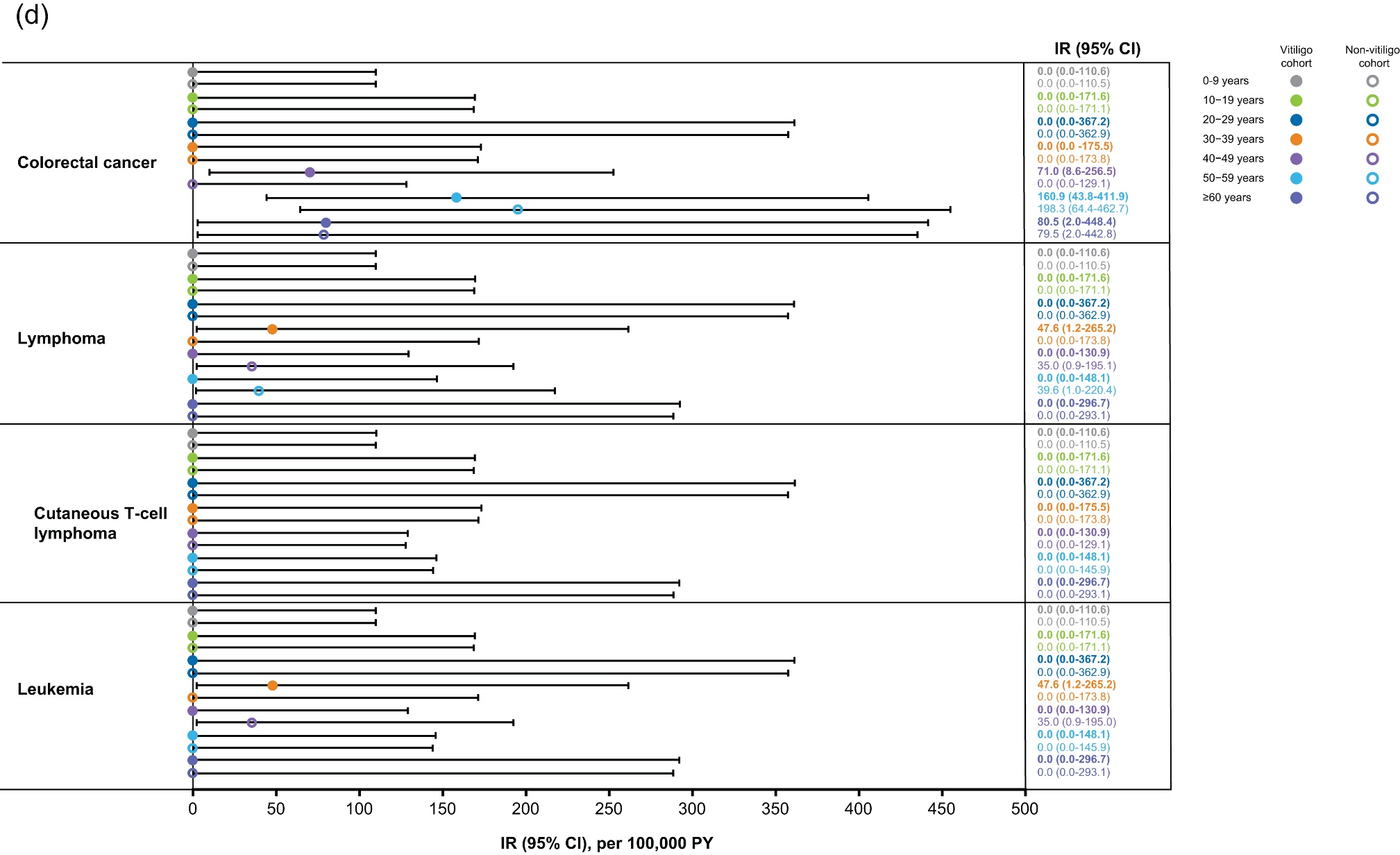


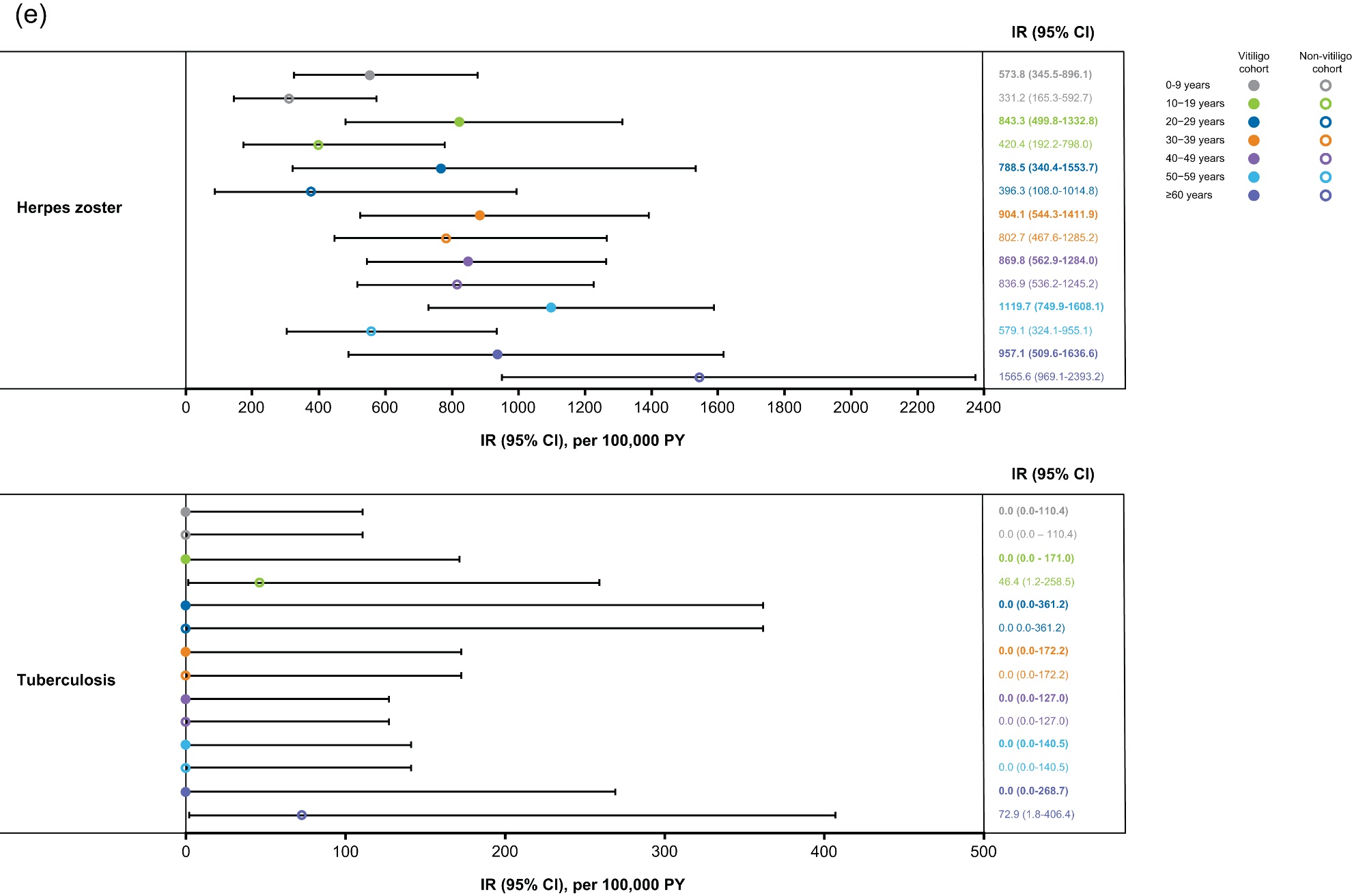

Supplement: Supplementary file 1 — Data S1. [file JDE-52-841-s001.docx]
